# A next-generation sequencing approach for high-resolution *S*-locus genotyping in apricot

**DOI:** 10.1038/s41598-026-59797-w

**Published:** 2026-08-26

**Authors:** Jorge Lora, Andrea Torres, José I. Hormaza, Javier Rodrigo, Afif Hedhly

**Affiliations:** 1 Instituto de Hortofruticultura Subtropical y Mediterránea La Mayora (IHSM La Mayora-CSIC-UMA), Algarrobo-Costa, 29750 Málaga, Spain; 2 Departamento de Ciencia Vegetal, Centro de Investigación y Tecnología Agroalimentaria de Aragón (CITA), Avda. Montañana 930, 50059 Zaragoza, Spain; 3 Instituto Agroalimentario de Aragón-IA2 (CITA-Universidad de Zaragoza), Zaragoza, Spain; 4 Aragonese Foundation for Research and Development (ARAID), 50018 Zaragoza, Spain; 5 Present Address: Estación Experimental de Aula Dei - Consejo Superior de Investigaciones Científicas (CSIC), Zaragoza, Spain

**Keywords:** Apricot, Self-incompatibility, *S*-locus genotyping by sequencing, *Prunus armeniaca*, Biotechnology, Genetics, Molecular biology, Plant sciences

## Abstract

Most temperate fruit crops exhibit a Gametophytic Self-Incompatibility (GSI) mechanism that prevents incompatible pollen tube growth and promotes outbreeding. In *Prunus* species, this system is governed by the multiallelic *S*-locus, which contains the *S*-haplotype-specific F-box (SFB) and *S-RNase* genes. Accurate determination of *S*-haplotypes is important for fruit breeding and orchard design and has traditionally relied on PCR-based analysis. However, PCR-based methods, combined with the partial sequencing of many *S*-alleles, may lead to ambiguous or incorrect allele identification. Next-generation sequencing (NGS) has generated numerous apricot genome datasets and revealed additional self-incompatibility alleles, yet *S*-locus genotypes remain unknown for many accessions. Here, we present a high-resolution NGS-based approach for *S*-locus genotyping based on genome filtering, mapping to a synthetic reference sequence, and automated *S*-allele calling. This approach is not intended to replace routine PCR-based *S*-genotyping, but rather to complement it in cases requiring sequence-level validation, clarification of ambiguous genotypes, or identification of previously uncharacterized alleles. Using this approach, *S*-haplotypes were inferred in 226 apricot cultivars, including 187 new genotype assignments, 30 confirmations of previously reported genotypes, and 9 cases that differed from previous reports. These results expanded the available information on pollination requirements to 422 apricot varieties. Sequence-based comparison of reported alleles documented 22 potential cases of synonymy and 20 cases of homonymy and supported the curation of 52 *S-RNase* and 28 *SFB* allele groups, increasing the number of reconstructed complete *S*-loci from 11 to 19. Furthermore, 129 cultivars were identified as carrying the *S*_c_ haplotype associated with self-compatibility. Overall, this study provides a high-resolution framework for apricot *S*-locus genotyping and a sequence-based resource to support future community efforts toward nomenclature harmonization.

**Supplementary Information:**

The online version contains supplementary material available at 10.1038/s41598-026-59797-w.

## Introduction

Apricot (*Prunus armeniaca* L.) is one of the most important temperate fruit crops worldwide, with world production reaching 4.6 million tons in 2024^1^. It belongs to the genus *Prunus* within the Rosaceae family, along with other economically significant stone fruit crops, such as peach (*P. persica* L.), Japanese plum (*P. salicina* Lindl.), European plum (*P. domestica* L.), sweet cherry (*P. avium* L.) and almond (*P. dulcis* (Mill.) D.A. Webb). China has historically been considered the center of origin of apricot, where several domestication events took place around 2000–3000 years ago. Apricots have traditionally been classified into six main groups according to their geographic origin: Dzhungar-Zailij, East Chinese, North Chinese, Middle-Asian, and European Iranian-Caucasian^2^. The European Iranian-Caucasian group is considered the most recent and the least variable, likely originating from a relatively small number of accessions, probably introduced through Armenia into Roman territories during the first century BCE. Apricot was indeed named Mela armeniaca (Armenian apple) by Dioscorides (around 50 CE) in Roman times^3^. Recent studies have identified northern Central Asia as the center of origin of the apricots grown in Europe, whereas Chinese cultivars trace their origin to southern Central Asia^4^.

Apricot and other stone fruits in the Rosaceae family have a GSI mechanism that inhibits incompatible pollen tube growth and promotes outbreeding. GSI is a widespread system among angiosperms, reported in species across 18 families^5^, and is believed to be the ancestral SI system for eudicots^6^. The GSI mechanism is controlled by a single *S*-locus, containing a multiallelic *S-RNase* acting as the female determinant, along with a male determinant that varies across genera and families. In *Prunus* species, the male determinant is a single multi-allelic F-box gene referred to as the *S* haplotype-specific F-box (*SFB*). In contrast, the male determinant consists of multiple F-box genes in both the Solanaceae family [referred to as *S* locus F-box (*SLF*)] and the subtribe Malinae within the Rosaceae family [referred to as *S* locus F-box brothers (*SFBB*)]^7,8^. The S-RNase protein, the female determinant, is secreted into the stylar transmitting tissue, while the F-box protein, encoded by the *SFB* gene, is found in the pollen tube. In heterozygous genotypes, where two different *S*-*RNases* are secreted into the style, both are taken up non-selectively by the pollen tube and both need to be detoxified to prevent RNA degradation and arrest of pollen tube growth in the style. The mechanisms of cytotoxicity and detoxification of *S*-*RNases* differ between *Prunus* species and the subfamily Malinae; in the latter it is suggested that the mechanisms resemble those described in the Solanaceae (reviewed in Matsumoto et al.^9^). The single *Prunus* SFB protein is suggested to be the trigger for self *S*-*RNase* cytotoxicity in the pollen tube, while a yet unidentified general inhibitor is hypothesized to play a detoxifying role^6^. Mutations in one of the two *S*-determinants have resulted in self-compatible genotypes in different *Prunus* species, including numerous apricot cultivars, most of them grown in Europe^10^. Additionally, a factor not linked to the *S*-locus, which causes self-compatibility when mutated, has also been identified in apricot^11^, further complicating the understanding of GSI.

The identification and characterization of self-incompatibility are major issues for stone fruit crop growers and breeders. Initially, methods to characterize the self-(in)-compatibility reaction involved hand pollination followed by observing pollen tube growth under a fluorescence microscope. To better characterize the specific *S*-haplotype, *S-RNase* alleles were subsequently identified by molecular and genetic approaches^12^. Since the identification of the *S*-locus-specific glycoprotein from *Brassica oleracea*^13^, PCR-based methods for *S*-allele genotyping have become available. Molecular approaches have confirmed early predictions^14^ of a high number of alleles at the *S*-locus. To date, 48 *S*-*RNase* alleles (*S*_1_ to *S*_20_, *S*_22_ to *S*_30_, *S*_52_, *S*_53_, *S*_93_ to *S*_107_, *S*_v_ and *S*_x_) and 32 *SFB* alleles (*SFB*_1–2_, *SFB*_4_, *SFB*_7–9_, *SFB*_11_, *SFB*_17_, *SFB*_23–27_, *SFB*_33_, _38, 41–42, 44–58_ and *SFB*_c_) have been identified in apricot^15–25^. Moreover, the NCBI database hosts 59 other unpublished *S*-*RNase* alleles and 27 *SFB* alleles.

In apricot, only a limited number of complete sequences of five *S*-*RNase* alleles (*S*_c_ DQ422947, *S*_*1*_ AY587561, *S*_*2*_ Ay587562, *S*_*4*_ AY587564, and *S*_*6*_/*S*_*52*_ KF951503) and five *SFB* alleles (*SFB*_1_, *SFB*_26_, *SFB*_33_, *SFB*_50_, and *SFB*_c_) have been reported^15,16,18,19,21,26^. While the *SFB* gene has a single, low length polymorphism intron in the 5’-UTR, the *S-RNase* gene has two introns with pronounced length polymorphisms. Consequently, *S*-allele identification relies mainly on PCR amplification of the *S-RNase* introns and, to a much lesser extent, on the *SFB* intron^12,18^. These PCR-based approaches remain the standard method for routine *S*-genotyping in apricot, particularly for breeding and germplasm characterization. To circumvent the low length polymorphisms of the *SFB* intron, semi-automated sequencing using fluorescently labeled primers, dot blot assays targeting hypervariable regions, or allele-specific hybridization using streptavidin-coated magnetic beads have been successful in other *Prunus* species, such as *P. avium*^27^ and Japanese plum^28,29^. While PCR methods remain the primary approach for *S*-allele identification in apricot, they may lead to ambiguous or inaccurate identifications of *S*-alleles due to the similar intron length in multiple alleles^18,30^. To avoid misidentification, sequencing of *S*-alleles becomes necessary, but the lack of full sequence data for many *S*-alleles, most of which have only been partially sequenced or have only their mRNA sequences available, may result in inconsistent or redundant allele names^30^. Nevertheless, incomplete sequencing of most *S*-alleles (some only for cDNA, *S-RNase* partial gene, or *SFB* fragments) can result in multiple *S*-alleles with the same name (homonymies) or the same allele with different names (synonymies), causing significant ambiguity in the identification of the *S*-alleles in apricot^11,30^. Furthermore, there are a large number of sequences on the NCBI website without any associated publication.

NGS has revolutionized genomic research by providing extensive genomic information, resulting in numerous plant genomes and transcriptomes, including for apricot^4^. However, many aspects, such as *S*-locus identification, remain largely unexplored. Rather than replacing current PCR-based techniques, NGS provides a significant opportunity to complement routine *S*-genotyping by enabling sequence-level validation, clarification of ambiguous cases, and the identification of previously uncharacterized alleles. Nonetheless, due to the high degree of polymorphism in the *S*-locus, its characterization on a simple reference genome is challenging, but new methodologies have been developed to address this issue^31–35^. These involve, among others, mapping genomic reads to synthetic reference sequences or filtering genomic reads with curated databases containing currently available *S*-locus sequences, although previous applications have generally focused on only one of the genes involved in the *S*-locus.

We recently improved *S*-locus characterization in Japanese plum (hybrids of *P. salicina* with other *Prunus* species) by including both the *SFB* and *S-RNase* genes^36^, thereby increasing consistency in sequence-based *S*-locus genotyping. Building on this framework, we present a high-resolution NGS-based approach for *S*-locus genotyping in apricot. This approach combines genomic read filtering using curated *Prunus S*-locus sequences, mapping to a synthetic *S*-locus reference, and automated Breadth-based allele calling. Applied to 226 cultivated apricot accessions, this workflow enabled large-scale *S*-haplotype inference, sequence-based comparison of previously reported alleles, reconstruction of additional *S*-haplotype relationships, and detection of the *S*_c_ haplotype associated with self-compatibility. Together, these analyses provide a robust sequence-based framework for *S*-locus genotyping, haplotype reconstruction, and the documentation of nomenclature inconsistencies that may inform future community-driven harmonization efforts.

## Results

### Curation of *S*-locus allele nomenclature

To clarify nomenclature inconsistencies, we compared reported *S*-*RNase* and *SFB* sequences from the literature and public databases. Historically established allele names were retained throughout the manuscript, and sequence similarities among previously reported alleles are presented as sequence-based relationships requiring further evaluation, rather than as formal renaming decisions. For grouping purposes in sequence comparisons, allele identifiers were assigned based on the earliest publication reporting the sequence or, when unavailable, the earliest submission to NCBI. This approach prioritizes nomenclatural stability while preserving traceability of historically used allele names.

In the case of self-compatible allele *S*_c_, sequence comparison showed that the *S*_c_- and *S*_8_-*RNase* alleles are identical, while the associated *SFB* gene at the *S*_c_-locus contains a characteristic insertion^37^. Accordingly, sequences corresponding to *S*_c_*-RNase* and *S*_8_*-RNase* were grouped into the *S*_8_/*S*_c_ allele group. However, divergence among sequences reported as *S*_8_*-RNase* revealed cases of homonymy, as the *S*_8_*-RNase* allele described in cultivar ‘Katy’^38^ differs from the *S*_8_/*S*_c_ sequence. To distinguish these sequence variants without proposing formal renaming, related groups were designated using suffixes (e.g. S_8_H^1^) (Supplementary Table S1).

**Fig. 1:**
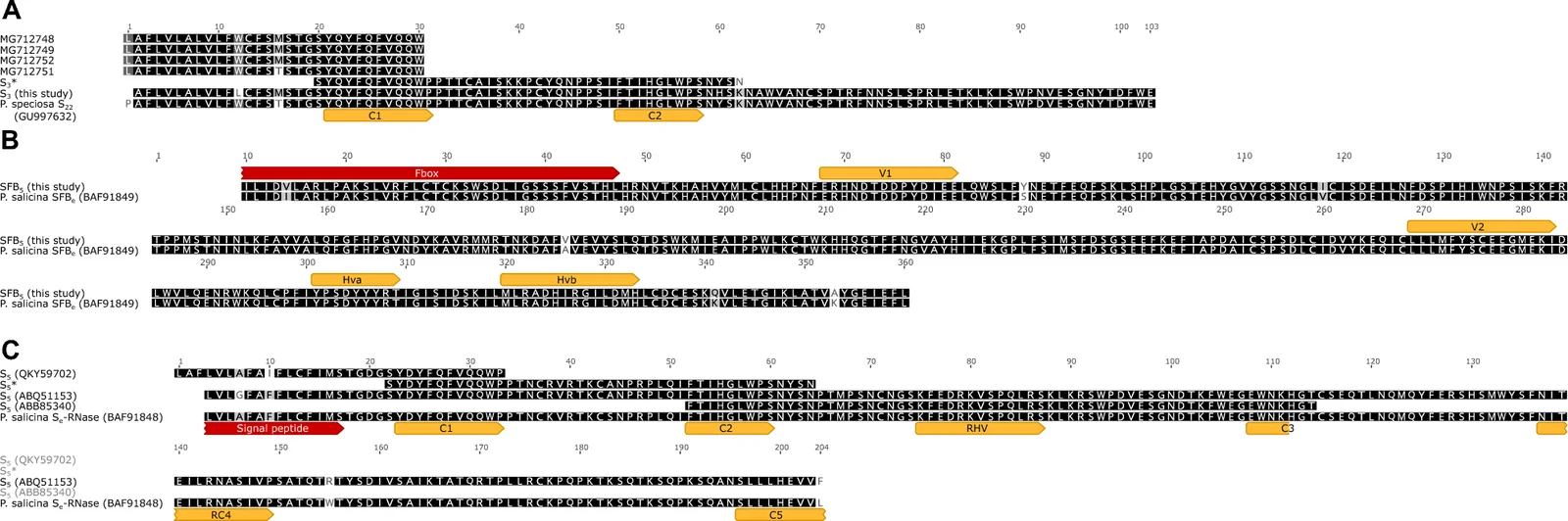
S-RNase and SFB alleles sequenced in this work. (**A**) Alignment of the deduced amino acid sequence of *S3-RNase* obtained in this study with the short *S3-RNase* sequence previously reported by Vilanova et al^39^. and with highly similar short sequences deposited in NCBI. Conserved regions C1 and C2 are indicated. (**B**) Alignment of the deduced amino acid sequence of SFB5 obtained in this study with the highly similar *SFBe* sequence of *P. salicina*. The F-box, variable regions, and hypervariable regions of SFB are indicated. (**C**) Alignment of deduced amino acid sequences previously associated with *S5-RNase*, together with the highly similar *Se-RNase* sequence of *P. salicina *and the short S5-RNase sequence reported by Vilanova et al^39^. The signal peptide, conserved regions, and hypervariable region of *S-RNases* are indicated. The *S5-RNase* sequences were grouped with the S13 allele group for sequence comparison while retaining historical allele identifiers in the manuscript and supplementary tables. Primer combinations SRc-F/Pru-C4R and PsSFB-F1/PsSFB-R1 were used for PCR amplification and Sanger sequencing of *S3-RNase* and *SFB5*, respectively (Supplementary Table S4).

The *S-RNase* alleles *S*_3_ and *S*_*5*_ had previously been inferred from fragment-based analyses but lacked full sequence characterization^39^. To clarify their identity, we cloned and sequenced *S*-locus fragments from selected cultivars. For *S*_3_, identical *S-RNase* sequences obtained from independent genotypes matched previously reported partial sequences, confirming correspondence with the historically recognized *S*_3_ allele (Supplementary Table S1, Fig. 1A). In contrast, comparative analysis of *S*_5_ revealed that its sequence is identical to the *S*_13_-*RNase* allele of *P. armeniaca* and shows high similarity to the *S*_*e*_*-RNase* of *P. salicina*, indicating that these sequences belong to a closely related trans-specific allele lineage (Fig. 1B-C). Accordingly, sequences previously associated with *S*_5_ were grouped with the *S*_13_ allele group for sequence comparison, with two distinct subgroups designated as *S*_13_H^1^ and *S*_13_H^2^ (Supplementary Table S1). Genotyping data further supported this relationship, as the corresponding *SFB* sequence co-segregates with the *S*_5_-*RNase* haplotype while clustering within the *S*_13_-related group (Supplementary Table S2). To preserve nomenclatural traceability, the *SFB* sequence was retained under the identifier *SFB*_5_, while explicitly noting its sequence relationship to the *S*_13_ allele group.

After curation, a small number of *S*-locus sequences could not be unambiguously assigned to previously described alleles, either because no corresponding identifier had been reported or because the sequence represented previously uncharacterized variants. To avoid introducing new formal nomenclature, these elements were assigned provisional identifiers (*S*_n_) for internal reference within this study (Table 1; Supplementary Table S1). These identifiers are not intended as formal allele designations but provide a consistent framework for sequence comparison.

**Table 1:** Grouped allele ID of *Prunus armeniaca SFB* and *S-RNase* sequences and first identification of the* S*-locus.

**Allele group**	**NCBI Reference sequence**		**Length (base pair)**	***S*-locus genotype identification**	
	**Submitted/edited names**	**ID nucleotide/cultivar**		**First time identified**	**Cultivar (genotype identified)**
*S*_1_	S1	AY587561/Goldrich	6879	1996	Goldrich (*S*_1_*S*_2_)
*SFB*_1_	SFB1	AY587563/Goldrich	1756	2004	Goldrich (*S*_1_*S*_2_)
*SFB*_1_H^1^	SFB1	FJ427210/Shanxing	405		
*S*_2_	S2	AY587562/Goldrich	6335	1996	Goldrich (*S*_1_*S*_2_)
*SFB*_2_	SFB2	AY587562/Goldrich	6335	2004	Goldrich (*S*_1_*S*_2_)
*S*_3_	SRN3	PP857343/Henderson	562	1996	Sunglo(*S*_2_*S*_3_)
*S*_4_		Genome/Chuanzhihong		1998	Harcot(*S*_1_*S*_4_)
*SFB*_4_		Genome/Chuanzhihong		2004	Harcot(*S*_1_*S*_4_)
*S*_6_		Genome/Sungold		1998	Moniqui (*S*_2_*S*_6_)
*SFB*_6_		Genome/Sungold			
*S*_8_/S_C_	CURHAP_LOCUS41163	Genome/Rojo pasion		1998	Colorao (*S*_5_*S*_c_)
*SFB*_8_/SFB_C_	CURHAP_LOCUS41164	Genome/Rojo pasion		2006	Currot (*S*_c_*S*_c_)
*S*_C_H^1^	Sc	MG891760/Palumella	352		
*S*_8_H^1^		Genome/Meihua			
*SFB*_8_H^1^		Genome/Meihua			
*S*_9_	S9	AY853594/Xinshiji	992	1996	Xinshiji (*S*_**9**_*S*_10_)
*SFB*_9_	SFB9	EU935588/Jiguang	1056		
*S*_10_	S10	DQ269997/Harmat	445	2005	Harmat (*S*_10_*S*_11_)
*S*_10H_1	S10	AY846872/Xinshiji	583		
*S*_11_	S11	EF173398/Harmat	565	2005	Harmat (*S*_10_*S*_11_)
*S*_11H_1	S11	DQ868316/Xinong 25	464		
*SFB*_11H_1	SFB11	EU652884/Chaoren	1134	2008	Chaoren (*S*_11_*S*_8_)
*S*_12_	S47	HQ342877/Keziaqia	575	2008	Chuanzhihong (*S*_12_*S*_14_)
*S*_12H_1	S12a	GU574200/Youyiyinbai	590		
*S*_13_	S13	DQ269995/Korai zamatos	1259	1998	Colorao(*S*_5_*S*_C_)
*SFB*_13_	SFB5	PP857342/Bulida	1055		
*S*_13_H^1^	S13	EF062341/Wardi	1251	2002	Beliana (*S*_7_*S*_c_)
*SFB*_13_H^1^	SFB13	EF062342/wardi	693	2014	
*S*_13_H^2^	S50	HQ342880/Suluke	659		
*SFB*_13_H^2^	SFB30	JN381946/Xiheiyexing	660	2014	Dongxing (*SFB*_41_)
*S*_14_	S66	JQ327152/Xiaobaixing	709	2008	Baixing (*S*_13_*S*_14_)
*SFB*_14_	SFB9	EF053404/Cegledi orias	1061		
*S*_15_	S15	DQ269999/Kech-pshar	665	2005	Kech-pshar (*S*_15_*S*_z_)
*S*_15H_	S15	DQ870631/Yingtiaojing	469	2006	
*S*_16_	S54	KT223013/Shushanggan	1296	2005	Zard (*S*_16_-)
*S*_16H_	S16	DQ870632/Duanzhixing	481	2008	Duanzhixing (*S*_16_*S*_18_)
*SFB*_16_H^1^	SFB27	FJ377728/Heiyexing	449		
*S*_17_	GBA52_018742	Genome/Yinxiangbai Xing		2008	Yinxiangbaixing (*S*_9_*S*_17_)
*SFB*_17_	GBA52_018743	Genome/Yinxiangbai Xing			
*S*_17H_	S17	EU516388/Satsuki	3127	2009	Chaoxian (*S*_24_*S*_27_)
*SFB*_17_H^1^	SFB	EU516388/Satsuki	3127	2009	Chaoxian (*S*_24_*S*_27_)
*S*_18_	S18	DQ870634/Duanzhixing	1337	2008	Duanzhixing (*S*_16_*S*_18_)
*SFB*_18_	SFB47	JN247793/Dabaiyouxing	434		
*S*_18H_1	S18	DQ270000/Kech-pshar	309		
*S*_19_	S51	HQ342881/Dongxing	768	2008	Baishaxing (*S*_13_*S*_19_)
*S*_20_		Genome/Stella		2008	Shuixing (*S*_14_*S*_20_)
*SFB*_20_		Genome/Stella			
*S*_23_	S53	KF975455/Saimati	1586	2009	Dafeng (*S*_17_*S*_23_)
*SFB*_23_	SFB50	HQ148074/Suogejia'nali	1313	2009	Dafeng (*S*_17_*S*_23_)
*SFB*_23_H^1^	S23	FJ377727/Xianhebao	466		
*S*_25_	S25	EU037264/Zhanggongyuan	994	2009	Zhanggongyuan (*S*_24_*S*_25_)
*SFB*_25_	SFB25	EU836686/Zhanggongyuan	1083	2009	Zhanggongyuan (*S*_24_*S*_25_)
*S*_26_	S26	EU037265/Huangkouwai	453	2009	Huangkouwai(*S*_11_*S*_26_)
*SFB*_26_	S26	EU652887/Huangkouwai	1128	2009	Huangkouwai(*S*_11_*S*_26_)
*SFB*_26_H^1^	S26	FJ377730/Youyiyinbai	504		
*SFB*_27_	S27	FJ377731/Erhuangxing	466		
*S*_28_	S28	EU836684/Chaoxianyihao	1353	2009	Chaoxian-1 (*S*_24_*S*_28_)
*S*_29_	S29	EF185300/Daguoxing	1076	2009	Daguoxing (*S*_29_*S*_30_)
*S*_30_	S30	EF185301/Daguoxing	405	2009	Daguoxing (*S*_29_*S*_30_)
*S*_30_H^1^	S30	LC431244/Yamagata No.3	684		
*S*_31_	S31	LC431245/Yamagata No.3	672		
*S*_33_	S33	LC431247/Heiwa	153		
*S*_35_	S35	GU574196/Yecanghua	624		
*S*_37_	S37	GU354240/Zhejiangxing	477		
*SFB*_38_	SFB38	JN247796/Akeyulvke	434	2014	Akeyulvke (*SFB*_38_)
*S*_40_	S40	GU354239/Xianhebao	752		
*S*_40_H^1^	S40	HQ342870/Kabakehuanna	572		
*SFB*_57_	S57	JN247794/Dashushanggan	434		
*S*_77_	S77	MG712754/GT390	318		
*S*_79_	S79	MG712756/AC103	296		
*S*_81_	S81	MG712758/DM101	380		
*S*_84_	S32	LC431246/Heiwa	321		
*S*_86_	S86	MG891761/KOSH269	402		
*S*_87_	S87	MG891762/GT390	398		
*S*_n_^1^	S	AY260450/Rival	345		
*S*_n_^2^	S	KY382359/AD1042 S	259		
*S*_n_^3^	New	Genome/Longwangmao			
*SFB*_n_^3^	New	Genome/Longwangmao			

See Table S1 for a full list of all accessions used in this study and references

Recently reported *S-RNase *alleles from large-scale genotyping studies (e.g. Zhang et al.^17^,) were considered in the context of sequence similarity and nomenclature. While these datasets expand the known diversity of *S*-alleles, differences in sequence coverage and naming conventions limit direct integration without reanalysis. Our approach therefore focuses on consistent cross-referencing of available sequences to document ambiguities while preserving established nomenclature.

During the curation process, several sequences previously annotated as *SFB* were identified as more divergent F-box-like genes and therefore not belonging to the *SFB* allele set (Supplementary Table S1). These sequences were excluded from the curated *SFB* allele dataset but retained for subsequent similarity analyses.

Taken together, we identified 52 *S-RNase* and 28 *SFB* allele groups, with multiple potential cases of synonymy (22) and homonymy (20) documented (see Supplementary Table S1). These curated allele assignments were based primarily on consistency with previously reported sequences and nomenclature, with sequence similarity used as a supporting criterion for identifying ambiguous cases. This curated dataset provides the foundation for subsequent analyses of sequence divergence and the structure of similarity relationships among *S*-locus alleles.

### Structure of sequence divergence among *S*-locus alleles

**Fig. 2:**
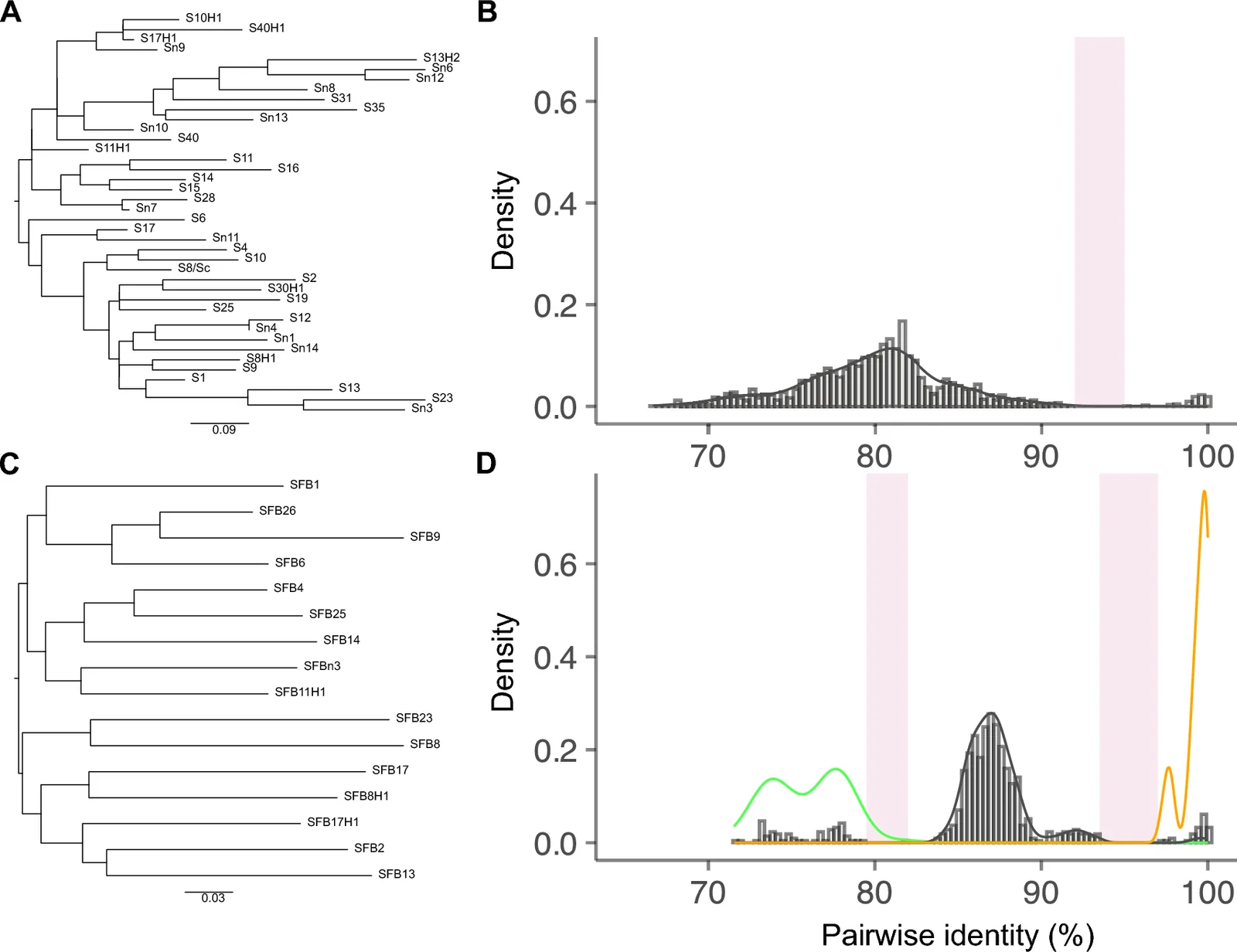
Phylogenetic relationships and pairwise sequence identity distributions for S-RNase and SFB sequences. (A–B) S-RNase sequences. (**A**) Phylogenetic tree reconstructed from a conserved region of the coding sequence using one representative sequence per allele group. This tree summarizes divergence among S-RNase allele groups and does not represent within-group variation. (**B**) Distribution of pairwise amino acid identity values calculated from the broader S-RNase sequence set for the same homologous region. Comparisons between sequences assigned to the same allele group form a narrow peak at high identity (> 95%), whereas comparisons between different allele groups form a broader distribution at lower identity values (~ 70–85%). A depletion of comparisons in the intermediate range (~ 90–95%; shaded) separates these regimes. (C–D) SFB sequences. (**C**) Phylogenetic tree reconstructed from coding sequences using one representative sequence per allele group, including the C-terminal variable/hypervariable region where available. This tree summarizes divergence among SFB allele groups. (**D**) Distribution of pairwise amino acid identity values calculated from the broader SFB/F-box sequence set. Three comparison regimes are observed: comparisons between SFB and more divergent F-box-like sequences (~ 70–78%; green fitted curve), comparisons among different SFB allele groups (~ 85–90%; grey/black fitted curve), and comparisons within the same SFB allele group or among highly similar records (> 95%; orange fitted curve). These regimes are separated by depletion zones (shaded), indicating discontinuities in sequence similarity. For panels B and D, histograms show pairwise identity values and smoothed density curves were fitted to the corresponding comparison classes to highlight the structure of pairwise similarity. Phylogenetic clustering is consistent with the identity distributions, with representative allele groups separated by substantial branch lengths and highly similar records captured by the high-identity regime in the pairwise distributions. Trees with branch support values are shown in Supplementary Figs. S1–S3.

Pairwise similarity analysis revealed that sequence identity among the curated S-RNase and SFB sequence sets is not uniformly distributed but instead shows a structured organization (Fig. 2). For S-RNase, analysis of the C-terminal coding region showed that comparisons between sequences representing different reported allele groups formed a broad distribution centered around ~ 70–85% identity, whereas comparisons within the same reported allele group were concentrated above ~ 95% (Fig. 2A–B, S-RNases). Notably, the distribution displays a marked depletion of comparisons in the intermediate range (~ 90–95%), separating highly similar sequence groups from the broader background of divergent comparisons. This pattern was consistently observed at both nucleotide and amino acid levels, suggesting that it is not simply driven by synonymous variation or alignment artefacts.

A more pronounced structure was observed for SFB sequences (Fig. 2C–D, SFBs). Pairwise identity distributions revealed a multi-modal pattern comprising three distinct regimes: a low-identity component (~ 70–78%) corresponding to comparisons between SFB and more divergent F-box proteins, an intermediate distribution (~ 85–90%) representing comparisons among distinct SFB allele groups, and a high-identity cluster (> 95%) associated with highly similar sequences within reported allele groups. These regimes are separated by clear depletion zones, indicating a non-uniform distribution of sequence similarity. Restricting the analysis to full-length coding sequences reduced the contribution of the low-identity component while preserving the separation between inter- and intra-group comparisons, supporting the interpretation that this structure reflects the organization of SFB sequence diversity rather than the inclusion of divergent F-box-like sequences alone.

To complement the analysis of pairwise sequence identity, phylogenetic trees were reconstructed using homologous aligned regions and a single representative sequence per allele group (Fig. 2A,C; Supplementary Fig. S1–S3). These trees summarized divergence among allele groups and were consistent with the identity distributions. For SFB, representative allele groups were separated by substantial branch lengths rather than forming clusters of near-identical specificities, in agreement with the inter-group identity regime around ~ 85–90%. Thus, the phylogenetic structure supports the separation between highly similar records within allele groups and the broader divergence observed among distinct *S*-locus specificities.

Together, these results indicate that pairwise identity values fall into distinct similarity regimes rather than forming a simple continuous gradient of divergence, particularly for SFB sequences. This structure provides a quantitative basis for distinguishing highly similar sequence groups from more divergent *S*-locus alleles within the curated dataset.

### Synthetic reference sequence of the *S*-locus

To construct a synthetic reference sequence of the S-locus, we selected, for each curated allele group, the accession containing the longest available nucleotide sequence (Table 1, Supplementary Table S1, and Supplementary Fig. S4). Whenever possible, complete *S*-haplotype sequences containing both *S-RNase* and *SFB* genes were used. In addition to previously published sequences, full *S*-haplotypes were extracted from reference genomes, allowing the inclusion of complete loci, including intergenic regions (Supplementary Table S3). The synthetic reference thus combines partial sequences from public databases with complete haplotypes derived from genome assemblies. We purposefully included the *S*_c_ haplotype, which corresponds to the self-compatible form associated with a transposon insertion within *SFB*_8_, enabling direct genotyping of self-compatibility associated with this haplotype.

Because many allele assignments were based on partial sequences containing only either *S-RNase* or *SFB*, we identified a set of “orphan” alleles for which haplotype relationships were unclear. To resolve these relationships, we integrated two complementary approaches. First, full *S*-haplotypes extracted from reference genomes were used to directly associate *S-RNase* and *SFB* sequences where possible. Second, we exploited co-occurrence patterns across the 226 genotyped accessions: allele pairs that consistently appeared together across multiple genotypes were inferred to represent components of the same haplotype. Using this approach, eight orphan *S-RNase*/*SFB* pairs were associated, corresponding to eight newly reconstructed haplotypes, thereby reducing the number of unlinked *S*-locus elements in the reference framework. As a result, previously unlinked *S-RNase* and *SFB* sequences could be represented as paired *S*-haplotype components, increasing the total number of reconstructed haplotypes to 19. In addition, this strategy also revealed previously unrecognized cases in which partial sequences assigned different identifiers likely correspond to the same allele. These cases were incorporated based on sequence similarity and co-occurrence patterns, and included in the final synthetic reference sequence. Together, these steps enabled the reconstruction of *S*-haplotypes from fragmented sequence data, integrating genomic and population-level information.

The final reference includes both complete *S*-haplotypes and curated partial sequences, providing a structured framework for subsequent genotyping analyses. The allele repertoire represented here reflects the currently available sequence diversity in public databases and literature but is not expected to be exhaustive, as additional *S*-alleles are likely to exist in under-sampled germplasm.

### *S*-locus genotyping and self-compatibility identification in 226 apricot cultivars

**Fig. 3:**
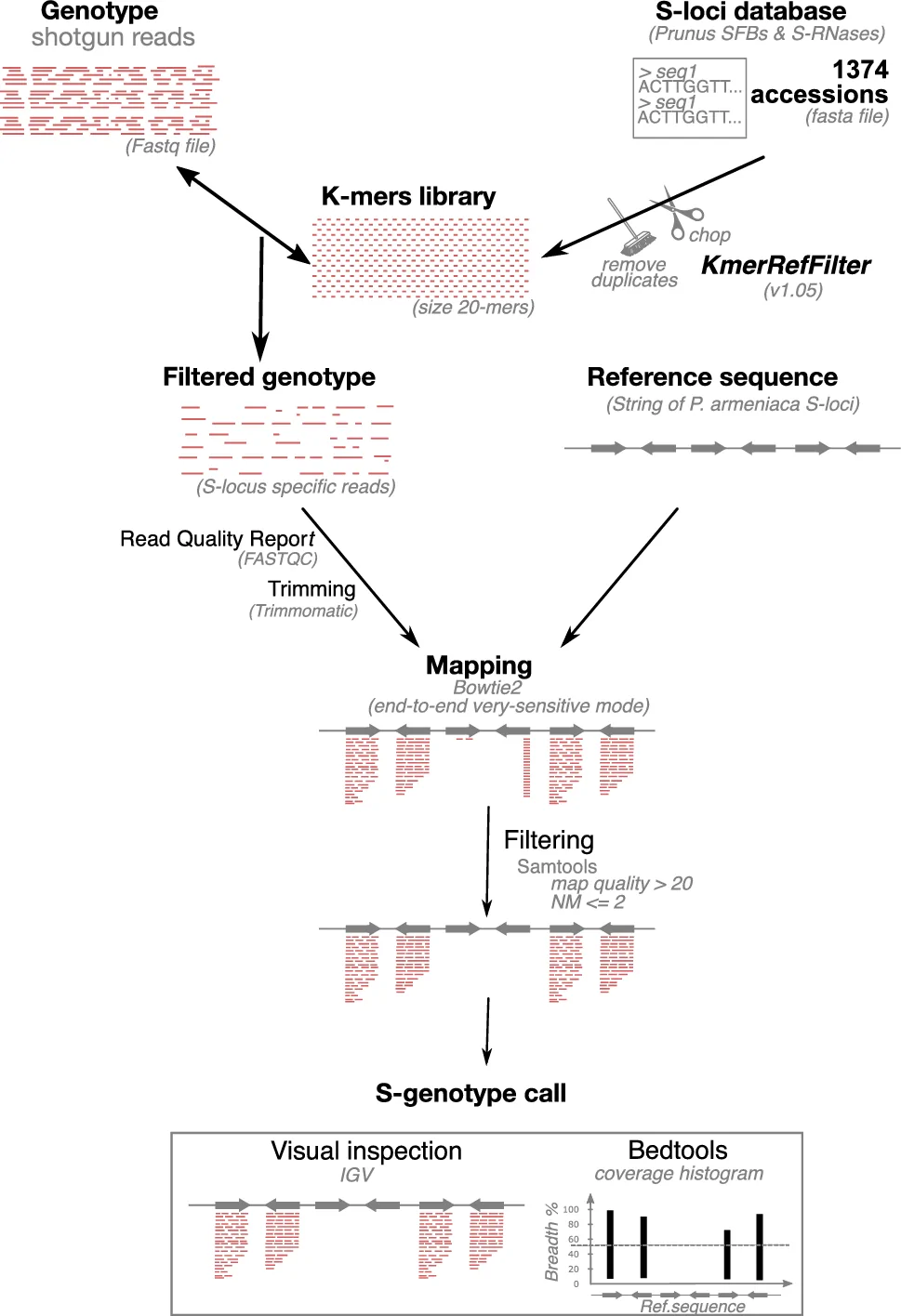
Bioinformatics pipeline for NGS-based high-throughput S-genotyping. Shotgun reads were first filtered using a Prunus S-locus k-mer database, then trimmed, mapped to the synthetic *P. armeniaca* S-locus reference, filtered by mapping quality and edit distance, and used for S-genotype calling based on Breadth of coverage. Breadth corresponds to the fraction of each reference gene covered by at least one mapped read.

Hundreds of apricot genomes are now publicly available, but the *S*-genotype is unknown for most accessions^4^. We used these resources to determine the *S*-genotypes of 226 cultivated apricot accessions by filtering whole-genome sequencing reads, mapping them to the synthetic *S*-locus reference, and applying an automated Breadth-based allele calling procedure. This command-line workflow was developed to scale our previous synthetic-reference approach, initially applied to Japanese plum and implemented through Galaxy^40^, to several hundred accessions. An initial k-mer-based filtering step, performed using a curated *Prunus S-RNase* and *SFB* sequence database and the k-mer filtering algorithm described by Genete et al.^31^, substantially reduced the amount of sequence data processed downstream. Whereas the previous workflow required approximately 3 h per accession, the command-line implementation reduced preprocessing time to less than 40 min per accession and allowed all 226 accessions to be processed in 6 days (Fig. 3).

A limitation of the filtering step is that reads derived from highly divergent or previously uncharacterized *S*-alleles may be under-recovered if the allele has little similarity to any *Prunus S*-locus sequence represented in the filtering database. In such cases, only one allele may be confidently identified. Alternatively, apparent single-allele genotypes may correspond to rare homozygous *S*-genotypes or to cases where the second allele is absent from the current reference, which cannot be distinguished using the present approach alone.

Using this pipeline, *S*-haplotypes were assigned to 226 apricot cultivars from New Zealand (2), China (18), Central Asia (45), India (2), Southern Africa (1), Northern Africa (11), Asia-Caucasus (16), Eastern Europe (12), Western Europe (84), and North America (35). Genotyping results were congruent with previously published genotypes for 30 cultivars, whereas nine cultivars showed differences from published *S*-genotypes, likely reflecting limitations of fragment-size-based PCR genotyping for some allele combinations. The specific case of ‘Sunglo’, genotyped from whole-genome data as *S*_c_*S*_9_ but identified by our Sanger sequencing as *S*_2_*S*_3_, may reflect the use of different plant materials in each case. All other Sanger-sequenced fragments confirmed the *S*-genotypes inferred by the NGS-based pipeline (Supplementary Fig. S5-S7).

The distribution of Breadth values across all allele mappings showed a clear separation between background mapping and positive allele calls (Supplementary Fig. S8). Most mappings exhibited very low Breadth values, whereas confidently assigned alleles clustered at high Breadth values, often close to complete coverage. Intermediate Breadth values were comparatively rare, and the 0.50 Breadth threshold used for allele calling fell within this low-density region, supporting its use as a conservative cutoff for distinguishing true allele presence from background mapping.

To evaluate the behavior of the pipeline when the synthetic reference was incomplete, selected *S*-locus genes were removed from the reference and accessions known to carry the removed alleles were remapped (Supplementary Fig. S9). The tested cases were chosen because closely related alleles remained present in the reference after removal of the target allele, representing a stringent test for false-positive reassignment. In particular, the *SFB* alleles selected for removal belonged to allele pairs showing high similarity within the inter-SFB range (~ 90–92%), where cross-mapping risk is expected to be highest. The tested cases included removal of the *S*_13_/*SFB*_5_ haplotype (*S*_13_ allele group), removal of the *S*_6_/*SFB*_6_ haplotype (*S*_6_ allele group), and simultaneous removal of the *S*_4_/*SFB*_4_ and *S*_23_/*SFB*_23_ haplotypes (*S*_4_ and *S*_23_ allele groups). In all cases, removal of the target allele(s) eliminated the corresponding high-Breadth signal, while retained true alleles remained detectable. No alternative allele exceeded the 0.50 Breadth threshold after removal of the target allele(s), indicating that the pipeline does not produce false-positive reassignment when the correct reference allele is absent.

To facilitate comparison with PCR-based genotyping, positions of commonly used S-RNase primer pairs were annotated in all available loci included in the synthetic reference (SRcF/SRc-R, Pru-T2/PruC2-R, Pru-C2/Pru-C6R, Pru-C2/PCE-R, EM-PC2/EM-PC3, and Pru-C2/Pru-C4R) and corresponding fragment sizes were predicted (Table 2; Supplementary Table S4). This analysis showed that, for several alleles, intron-length differences are below the resolution expected from standard agarose gel electrophoresis, and that the use of both introns is required to distinguish some allele combinations.

**Table 2:** Expected PCR fragment sizes (base pair) for various primer combinations commonly used in *P. armeniaca* to amplify *S-RNase* alleles.

**Allele group**	**Intron 1**		**Intron 2**			
	**SRcF/SRc-R**	**Pru-T2/PruC2-R**	**Pru-C2/Pru-C6R**	**Pru-C2/PCE-R**	**EM-PC2/EM-PC3**	**Pru-C2/Pru-C4R**
*S*_1_	400	483	2109	2143	2165	2261
*S*_2_	327	413	839	873	895	991
*S*_3_	267	350	211	245	*no.seq*	*no.seq*
*S*_4_	260	346	301	335	357	453
*S*_6_	421	507	1239	1273	1295	1391
*S*_8_	353	439	2805	2839	2861	2957
*S*_8_H^1^	354	437	540	574	596	692
*S*_9_	*no.seq*	*no.seq*	601	635	657	753
*S*_10_	*no.seq*	*no.seq*	*no.seq*	*no.seq*	*no.seq*	*no.seq*
*S*_10_H^1^	*no.seq*	*no.seq*	210	244	266	362
*S*_11_	302	382	*no.seq*	*no.seq*	*no.seq*	*no.seq*
*S*_11_H^1^	*no.seq*	*no.seq*	408	442	464	560
*S*_12_	*no.seq*	*no.seq*	304	330	360	456
*S*_12_H^1^	*no.seq*	*no.seq*	542	576	598	*no.seq*
*S*_13_	394	480	778	812	834	*no.seq*
*S*_13_H^1^	377	460	1201	1235	1257	1353
*S*_13_H^2^	*no.seq*	*no.seq*	345	379	401	497
*S*_14_	*no.seq*	*no.seq*	435	469	491	587
*S*_15_	*no.seq*	*no.seq*	607	641	663	759
*S*_15_H^1^	*no.seq*	*no.seq*	413	447	469	*no.seq*
*S*_16_	*no.seq*	*no.seq*	1024	1058	1080	1176
*S*_16_H^1^	*no.seq*	*no.seq*	425	459	481	*no.seq*
*S*_17_	221	310	431	465	487	583
*S*_17_H^1^	*no.seq*	*no.seq*	363	397	419	515
*S*_18_	379	*no.seq*	1281	1315	1337	*no.seq*
*S*_18_H^1^	*no.seq*	*no.seq*	251	285	307	*no.seq*
*S*_19_	*no.seq*	*no.seq*	490	524	546	642
*S*_20_	419	505	1871	1904	1926	2022
*S*_23_	338	421	639	673	695	791
*S*_25_	*no.seq*	*no.seq*	716	750	772	861
*S*_26_	*no.seq*	*no.seq*	419	453	475	*no.seq*
*S*_28_	*no.seq*	*no.seq*	1076	1115	1137	1233
*S*_29_	*no.seq*	*no.seq*	1089	1123	1145	*no.seq*
*S*_30_	*no.seq*	*no.seq*	418	452	474	*no.seq*
*S*_30_H^1^	*no.seq*	*no.seq*	*no.seq*	*no.seq*	*no.seq*	*no.seq*
*S*_31_	*no.seq*	*no.seq*	*no.seq*	*no.seq*	*no.seq*	*no.seq*
*S*_33_	*no.seq*	*no.seq*	*no.seq*	*no.seq*	*no.seq*	*no.seq*
*S*_35_	*no.seq*	*no.seq*	256	290	312	408
*S*_37_	*no.seq*	*no.seq*	*no.seq*	*no.seq*	*no.seq*	*no.seq*
*S*_40_	*no.seq*	*no.seq*	483	517	539	635
*S*_40_H^1^	*no.seq*	*no.seq*	297	327	353	445
*S*_77_	318	*no.seq*	*no.seq*	*no.seq*	*no.seq*	*no.seq*
*S*_79_	296	*no.seq*	*no.seq*	*no.seq*	*no.seq*	*no.seq*
*S*_81_	380	*no.seq*	*no.seq*	*no.seq*	*no.seq*	*no.seq*
*S*_84_	408	494	*no.seq*	*no.seq*	*no.seq*	*no.seq*
*S*_86_	402	*no.seq*	*no.seq*	*no.seq*	*no.seq*	*no.seq*
*S*_87_	398	*no.seq*	*no.seq*	*no.seq*	*no.seq*	*no.seq*
*S*_n_^1^	*no.seq*	*no.seq*	*no.seq*	*no.seq*	*no.seq*	*no.seq*
*S*_n_^2^	352	*no.seq*	*no.seq*	*no.seq*	*no.seq*	*no.seq*
*S*_n_^3^	405	488	2804	2844	2866	2962
*S*_c_	258	*no.seq*	*no.seq*	*no.seq*	*no.seq*	*no.seq*

The synthetic reference also enabled genotyping of self-compatibility associated with the *S*_c_ haplotype. Because *S*_c_ haplotype is characterized by a FaSt MITE insertion within the *SFB* gene, the insertion sequence was included in both the filtering database and synthetic reference. Highly similar FaSt-related MITE sequences occur throughout *Prunus* genomes: previous work identified 121 full copies in peach, and our BLAST searches detected 45–103 full copies with > 94% pairwise identity in *P. armeniaca*, *P. mandshurica*, and *P. sibirica* reference genomes (Supplementary Table S5). Consequently, reads originating from dispersed genomic copies of the MITE can map to the *S*_c_-associated insertion, even in accessions lacking *S*_c_. For this reason, *S*_c_ genotyping was based not on insertion coverage alone, but on read continuity across the junction between the SFB coding sequence and the MITE insertion.

**Fig. 4:**
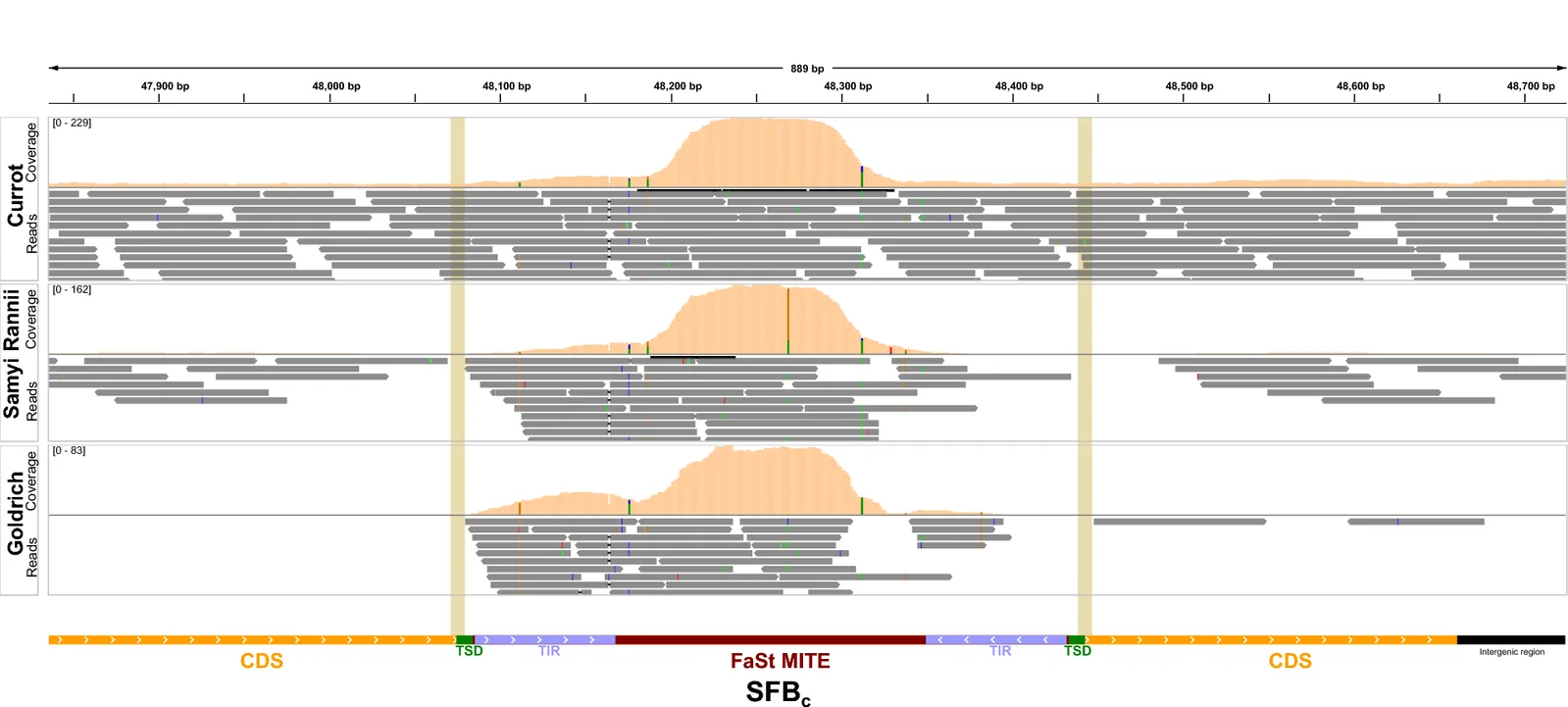
Genotyping of Sc-associated self-compatibility. Detail of read alignment showing coverage histograms (colored light brown) and mapped reads (grey boxes) in the region encompassing the FaSt MITE insertion within the coding sequence of the SFB gene in the S_c_ haplotype. Three representative apricot accessions are shown: ‘Currot’, carrying the S_c_-associated mutant SFB allele, ‘Samyi Rannii’, carrying the wild-type S_8_ haplotype, and ‘Goldrich’, which carries neither S_8_ nor S_c_. Colored rectangles within reads indicate bases that differ from the reference sequence. The relative size and colors in the multicolored coverage bars indicate the mismatch frequency of each base at that position. The figure is an edited IGV snapshot. CDS: coding sequence; TIR: terminal inverted repeat; TSD: target site duplication.

This distinction is illustrated by representative read alignments (Fig. 4). In ‘Samyi Rannii’, which carries the wild-type *S*_8_ haplotype, reads mapped to the MITE insertion but did not span the SFB–MITE junction, indicating that these reads originated from other genomic copies of the transposon. In contrast, in ‘Currot’ (*S*_c_*S*_c_), read coverage was continuous across the SFB–MITE junction, confirming the presence of the *S*_c_-associated insertion. The absence of junction-spanning reads in ‘Goldrich’ (*S*_1_*S*_2_) further confirmed that MITE coverage alone is insufficient for *S*_c_ assignment. PCR and Sanger sequencing analyses of eight cultivars known to carry the *S*_c_-associated mutant *SFB* allele, using *SFB*- and insertion-specific primers^41^, confirmed the presence of both the *SFB*_8_ allele and transposon-specific fragments (Supplementary Fig. S5-S7).

**Fig. 5:**
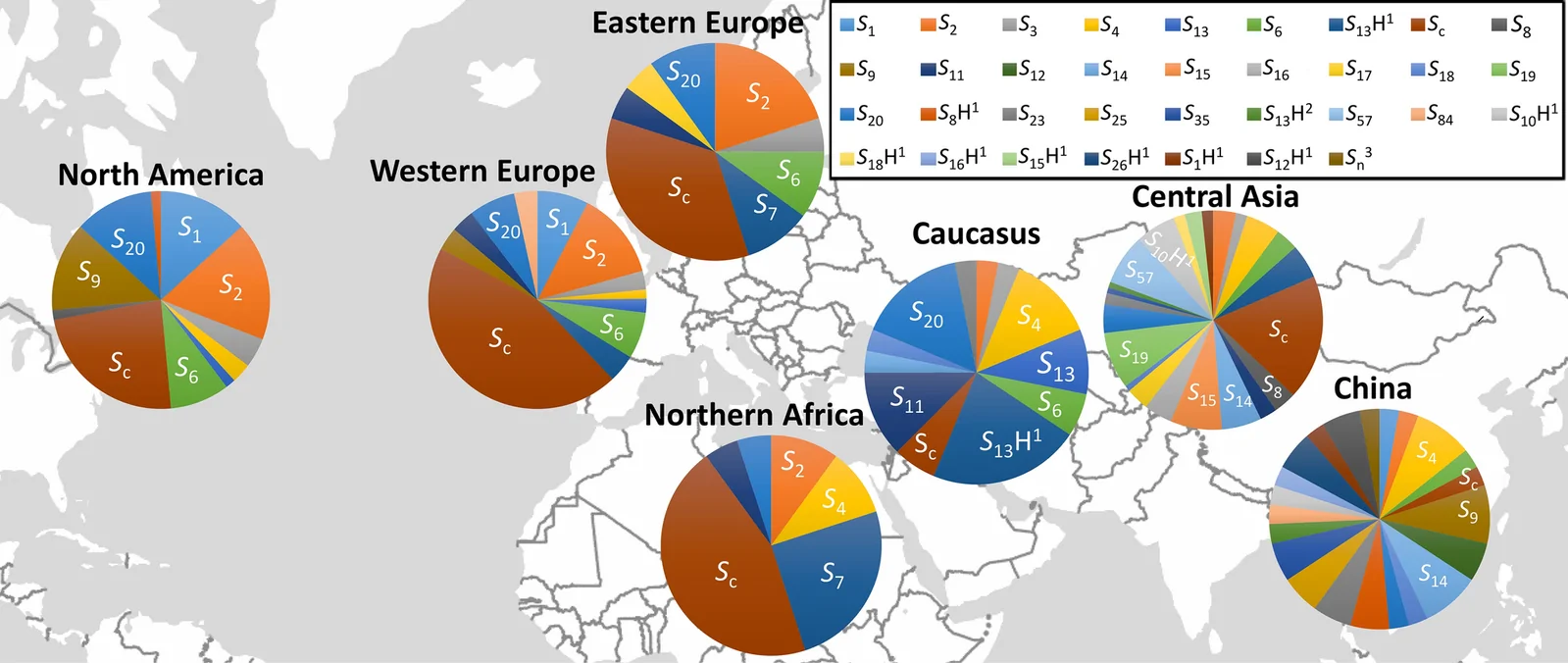
Geographical distribution of S-haplotypes in apricot accessions. The grouped allele ID of each S-locus is labelled and color-coded in the upper right square. In the circles, only grouped allele IDs with an allele frequency greater than 6% are shown. The largest number of S-loci is observed in China and Central Asia, with a decrease in their number in Western regions. *Sc* is the most abundant haplotype and has the broadest distribution, whereas other S-loci, such as *Sc*12H^1^ and S_n_^3^, have a more limited distribution and were found only in China. Central Asia includes Afghanistan, India, Kazakhstan, Kyrgyzstan, Pakistan and Uzbekistan. Caucasus includes Armenia, Azerbaijan and Iran. Northern Africa includes Morocco and Tunisia. Eastern Europe includes Hungary, Romania, Slovakia and Ukraine. Western Europe includes France, Greece, Italy and Spain. North America includes Canada and USA. Map tiles by Stamen Design, licensed under CC BY 4.0. Data from OpenStreetMap, under ODbL.

*S*_c_ was the most frequent haplotype in the dataset, detected in 129 cultivars (57% of 226 accessions). The number of *S*-haplotypes was highest in accessions from China (22) and Central Asia (23), and decreased westward, with 12 haplotypes identified in Caucasian countries, 8 in Eastern Europe, 12 in Western Europe, 6 in North Africa, and 10 in North America (Fig. 5; Supplementary Table S6). Because of the low number of cultivars from India (2), New Zealand (2), and Southern Africa (1), these regions were not considered for regional allele-frequency comparisons.

## Discussion

### A high-resolution *S*-genotyping framework based on whole-genome sequencing

Inconsistencies in *S*-locus IDs within *P. armeniaca* arise from the use of diverse primer pairs and reliance on un-sequenced or partially sequenced alleles. Accurate *S*-allele identification should cover most coding regions and hypervariable segments. While Sanger sequencing remains the standard for small-scale projects, the availability of whole-genome sequencing datasets provides an opportunity for sequence-level *S*-genotyping in large germplasm collections^4^. However, the extreme polymorphism of the *S*-locus makes direct mapping to a single reference genome poorly suited for this purpose.

Synthetic-reference and k-mer-filtering strategies have been used successfully for *S*-locus genotyping in other taxa, including *Malus domestica* and *Arabidopsis halleri*^31,34^. Our previous work in Japanese plum extended this synthetic-reference approach by incorporating both *S*-*RNase* and *SFB* sequences^35^. In the present study, we adapted the k-mer filtering strategy of Genete et al.^31^ using a curated *Prunus S-RNase*/*SFB* database, making the workflow scalable to hundreds of apricot genomes while retaining sequence-level resolution.

In contrast to the *A. halleri* framework, which was designed for de novo allele discovery from an intentionally incomplete SRK reference, our implementation was adapted here for conservative *S*-locus genotyping in apricot. The k-mer filtering step was used primarily to enrich reads with similarity to known *Prunus S*-locus sequences before mapping to the curated apricot synthetic reference. Including *S*-locus sequences from multiple *Prunus* species takes advantage of trans-specific conservation among *S*-allele lineages and reduces the risk of discarding reads from incompletely characterized apricot regions, while also substantially reducing the amount of genomic data processed downstream. Incorporating both *S*-determinants in the synthetic reference allowed genotype calls to be interpreted at the *S*-haplotype level, providing an internal consistency check between the female and male components of the locus.

However, classical PCR-based approaches remain the fastest and most cost-effective method for routine *S*-genotyping in apricot, particularly when intron length polymorphisms or *S*_c_-diagnostic primers are used. Accordingly, the NGS-based approach presented here is not intended to replace these routine methods, but rather to serve as a high-resolution tool for resolving ambiguous genotypes, detecting previously uncharacterized alleles, reconstructing complete *S*-haplotypes, and documenting sequence relationships consistent with potential synonymy or homonymy.

### Documenting redundancies and inconsistencies in apricot S-locus sequences

To date, a total of 48 *S-RNase* and 32 *SFB* alleles of *P. armeniaca* have been published^15–18^. However, additional *S-RNase* and *SFB* alleles are listed in the NCBI database, with identifiers as high as 107 for S-RNase and 60 for SFB. Recent studies based on PCR amplification and sequencing of *S-RNase* fragments have further expanded the catalogue of reported alleles, particularly in Chinese germplasm^17^. Because allele identification has historically relied on partial sequences and independent naming across studies, inconsistencies in allele designation have accumulated over time. Sequence-based comparison across datasets therefore remains important to identify cases where different names may correspond to highly similar sequences and to facilitate future harmonization of *S*-allele nomenclature.

For example, our analysis supports the previously suggested relationship between the *S6-* and *S52*-RNase sequences^30^. Conversely, some allele names have been assigned to divergent sequences, as illustrated by sequences previously reported under the *S*_9_-*RNase* designation. Together, these examples illustrate how independent naming based on partial sequences can generate apparent synonymies and homonymies. Similar nomenclature inconsistencies have also been reported in other *Prunus* species, including Japanese plum^36,41,42^, and in other Rosaceae fruit crops such as European pear^43^. The application of our curation and genotyping methodology to 226 apricot cultivars helped document sequence relationships and inconsistencies, resulting in a curated set of 52 *S-RNase* and 28 *SFB* allele groups.

Misidentification of *S*-alleles in *P. armeniaca* also affects the assignment of cultivars into incompatibility groups, which are traditionally based on PCR fragment size polymorphisms rather than sequence analysis. These groups have been reported by different research groups using partly different PCR-based approaches^22,23,30,44–47^. While the first incompatibility groups have been consistently reported and later confirmed through sequence analyses, several *S*-alleles included in other groups remain unverified by sequence data. For example, alleles reported as *S*_19_ have been associated with different PCR fragment sizes across studies, and the available sequence information is insufficient to determine whether all these reports refer to the same allele^44,47,48^. Such inconsistencies indicate that incompatibility groups should be re-evaluated with sequence information whenever possible.

The long-term accumulation of *S*-allele names in apricot, often derived from partial sequences, different primer sets, or independent regional studies, has resulted in inconsistencies that complicate comparisons across datasets. In this context, our analysis aims to provide a structured overview of *S*-locus sequence relationships by compiling available *S*-locus sequences and quantifying their similarities. By presenting these relationships in a common comparative framework, our results provide a resource that may facilitate future community-based efforts toward harmonization of *S*-allele nomenclature, while preserving the historical identifiers currently in use.

### Genotype-based reconstruction of orphan *SFB*s and *S-RNases* into *S*-haplotypes

In apricot, paired *S-RNase* and *SFB* sequences from the same *S*-haplotype were first made available in 2004 (*S*_1_, *S*_2_ and *S*_4_^21^). Additional *S*-haplotypes were described over the following six years, including *S*_8_, *S*_c_, *S*_22_ (included in the *S*_8_H^1^ allele group)_,_* S*_17,_ *S*_27_ (included in the* S*_17_H^1^ allele group)_,_ *S*_23,_ *S*_25_ and *S*_26_, although many of these records contained partial gene sequences and limited information on the intergenic region^15,19,37,44^. After curation, these haplotypes retained their historical identifiers. Since then, few additional complete *S*-haplotype sequences have been available, and much of the remaining information consists of orphan *S-RNase* or *SFB* sequences, sequences affected by nomenclature ambiguity, or unannotated haplotypes that can be extracted from reference genomes.

Most available sequences originate from PCR products generated in *S*-genotyping studies based on fragment length polymorphism. Because the *S-RNase* gene contains two highly polymorphic introns, whereas *SFB* has a single, short, and less polymorphic intron upstream of the translation start site, *S*-genotyping studies have historically focused more strongly on *S-RNase*. This is reflected in the available sequence databases, with 178 accessions corresponding to 102 *S-RNase* alleles, compared with 81 accessions corresponding to 47 *SFB *alleles in NCBI GenBank.

This imbalance likely contributes to the accumulation of orphan *S*-locus elements and to the limited number of complete *S*-haplotypes available. Combining sequence curation with preliminary genotyping of 226 apricot cultivars using a first draft of the synthetic reference allowed us to associate 16 orphan *S*-locus elements into 8 additional *S-RNase*/*SFB* haplotype pairs. This brings the total number of reconstructed *S*-haplotypes to 19.

All *Prunus S*-haplotypes characterized so far contain *SFB* and *S-RNase* genes arranged in head-to-head orientation, with the exception of the *S*_2_ locus, in which both genes are in head-to-tail orientation^49^, without any apparent effect on the self-incompatibility response^21^. Based on available complete *S*-loci of *P. armeniaca*, the intergenic region between *SFB* and *S-RNase* ranges from 940 to 8175 bp, depending on the *S*-haplotype. Variation in intergenic region size has also been reported in other *Prunus* species^49^, reaching up to 41 kb in *P. salicina*^50^.

Complete haplotype reconstruction also provides a framework for examining broader *S*-locus organization, including variation in intergenic regions and the presence of neighboring *SFB*-like genes. Although these neighboring F-box genes may be relevant to models of non-self *S-RNase* detoxification, their functional contribution in *Prunus* remains unresolved.

### Global diversity of *S*-alleles and predominance of *Sc*-associated self-compatibility

The geographic distribution of *S*-alleles in 226 apricot cultivars showed a gradual decrease in their number from China (22) and Central Asia (23) to Europe (6–12). High allelic diversity in Chinese cultivars is consistent with the long-standing hypothesis that this region contributed to apricot diversification^3^. The slightly higher number of alleles detected in Central Asia is also consistent with recent genomic evidence suggesting that European and Chinese apricot cultivars originated from wild populations in northern and southern Central Asia, respectively^4^. This progressive westward decline in *S*-allelic diversity is consistent with previous apricot genetic diversity studies^18,51,52^ and may reflect domestication bottlenecks, as observed in other fruit crops^53,54^. Another contributing factor may be the intentional selection of self-compatible cultivars, which can lead to increased inbreeding and further reduction in genetic diversity^55^. Indeed, the *S*_c_ haplotype predominated in the genotyped apricot cultivars (57%, n = 226), especially in cultivars grown in Western Europe (77%, n = 84). The European apricot group is considered one of the most recent and least variable groups and consists largely of self-compatible cultivars, although the introduction of new cultivars carrying sharka resistance has increased the number of self-incompatible cultivars in Europe^18^. Although Sc has previously been reported mainly in European germplasm and was not detected in a recent survey of Chinese cultivars^17^, our dataset identified a single accession carrying this haplotype. Given the potential importance of this observation, independent validation will be necessary to confirm its occurrence and origin.

The *S*_c_ haplotype is thought to have originated from the *S*_8_ haplotype through the insertion of a MITE named FaSt within the coding region of the *SFB* gene^37,56^. Recently, another MITE insertion has also been reported as a causative factor for self-compatibility in *Citrus hindsii*^57^. In *P. armeniaca*, the FaSt MITE insertion has been proposed to have occurred in southeastern Turkey, where the *S*_8_ haplotype has been observed among local cultivars^56^. Although our dataset did not include cultivars from Turkey, the *S*_8_ haplotype was detected in cultivars from neighboring regions, including three from Kazakhstan and one from Uzbekistan, and in the ‘Sweet Cot’ cultivar grown in the USA. The current version of the synthetic reference sequence does not distinguish *S*_c_ from the related *S*_c_ variant recently reported in some Moroccan cultivars, which carries a second FaSt insertion nested within the original insertion^56^. Including this nested insertion in future versions of the synthetic reference could allow discrimination between these *S*_c_-associated haplotypes, in the same way that junction-spanning reads allowed us to distinguish *S*_8_ from *S*_c_. In addition, although self-compatibility in apricot has been mainly associated with the FaSt insertion, a mutation at the *M*-locus, unlinked to the *S*-locus, has also been shown to break the self-incompatibility reaction in this species^11^. Recently, an HRMA-based marker-assisted selection approach has been developed for the identification of self-compatibility associated with the *S*- and *M*-loci^41^.

## Conclusion

In this work, we used a whole-genome-sequencing-based approach to infer *S*-haplotypes in 226 apricot cultivars, including 187 new genotype assignments, 30 confirmations of previously reported genotypes, and 9 cases that differed from earlier PCR-based reports. Together with previously published data, these results expand the available information on pollination requirements for apricot cultivars.

By compiling *S-RNase* and *SFB* sequences from public databases, published studies, and available reference genomes, we generated a curated synthetic *S*-locus reference for apricot genotyping. This resource enabled the identification of 52 *S-RNase* and 28 *SFB* allele groups, the reconstruction of additional *S*-haplotype relationships from orphan *S-RNase* and *SFB* sequences, and the documentation of sequence relationships consistent with potential synonymy or homonymy among previously reported alleles.

The pipeline also enabled detection of the *S*_c_ haplotype associated with self-compatibility and can be extended in future work to distinguish additional self-compatibility-associated mutations, including the nested FaSt insertion and *M*-locus-associated self-compatibility. Importantly, this approach is not intended to replace routine PCR-based genotyping, but rather to complement it in cases requiring sequence-level validation, clarification of ambiguous genotypes, or characterization of previously unrecognized *S*-locus variation.

Overall, our results provide a sequence-based resource for apricot *S*-locus genotyping and a transparent framework for comparing reported *S*-locus sequences. While formal harmonization of *S*-allele nomenclature should remain a community-driven process, the curated relationships reported here may contribute to future standardization of S-allele classification.

## Materials and methods

### Sequence curation and synthetic *S*-locus reference assembly

To document sequence relationships and nomenclature inconsistencies among reported apricot *S*-locus sequences, we first performed a systematic search for *P. armeniaca S-RNase* and *SFB* sequences in NCBI and in the scientific literature. This search retrieved *S-RNase* sequences reported under 87 allele designations from 163 accessions, and *SFB* sequences reported under 47 allele designations from 81 accessions, however, the most recently published accessions were not included in the analysis^17^.

Using short conserved *P. armeniaca S*-locus sequences, we also performed BLASTN searches against the nine available apricot reference genomes: ‘GSYX’, ‘Meihua’, ‘Rojo Pasión’ and ‘Yinxiangbai Xing’ hosted at NCBI, and ‘Chuanzhihong’, ‘Longwangmao’, ‘Marouch n14’, ‘Stella’ and ‘Sungold’ hosted at the Genome Database for Rosaceae^58^. Complete *S*-locus sequences were retrieved when available, including S-RNase, SFB, intergenic regions and flanking sequences where possible (Supplementary Table S1; accession numbers in Supplementary Table S5).

Multiple sequence alignments were generated separately for *S-RNase* and *SFB* sequences. Pairwise nucleotide and amino-acid identities were calculated from homologous aligned regions to evaluate sequence similarity among reported allele groups. Because available sequences differed in length and completeness, phylogenetic analyses were performed both on broader alignments and on homologous regions shared among sequences. Where appropriate, representative sequences were used to avoid overrepresentation of highly similar records. For S-RNases, trees were reconstructed using the full amino-acid alignment and one shorter homologous region of 95 amino acids covering the hypervariable region. For SFB, trees were reconstructed using both an alignment of longer near-complete sequences and a broader alignment including shorter available sequences.

Phylogenetic reconstruction was performed with IQ-TREE v2.3.6 using maximum likelihood inference, ModelFinder automatic model selection (-m MFP), 1,000 ultrafast bootstrap replicates (-B 1000), and SH-aLRT branch tests (-alrt 1000). Analyses were run with automatic thread selection (-T AUTO). For sequence curation, amino-acid identity values above 96% were used as an operational criterion to flag highly similar records for grouping and manual inspection. This threshold was not interpreted as evidence of functional equivalence or as a formal nomenclatural decision. Final grouping decisions were evaluated in the context of sequence alignments, phylogenetic position, historical naming, sequence coverage, and co-occurrence of S-RNase/SFB calls where available.

Sixteen records previously annotated as SFB (HQ148064, HQ148070, HQ148071, HQ148073, HQ148076, HQ148065, HQ148069, JN381950, HQ148077, HQ148078 and JN381948) or S-RNase (AF468675, AF468678, GU574201, MF685202 and MF685203) were excluded from the curated *S-RNase/SFB* allele sets because they did not cluster with the curated SFB or S-RNase sequence sets.

The synthetic reference sequence was assembled by concatenating curated *S*-locus sequences into a single synthetic reference (Supplementary Fig. S4). Depending on the sequence available for each locus, the reference included complete *S*-locus regions extracted from reference genomes, individual *S-RNase* or *SFB* sequences, and orphan *S*-locus elements for which only one haplotype component was available. We refer to orphan *S*-locus elements as cases in which only one of the two expected components of the haplotype, *S-RNase* or *SFB*, was available. When different sequences covered non-overlapping regions of the same gene, consensus sequences were generated and artificial N-spacers were inserted where needed to represent non-contiguous fragments. In other cases, consensus sequences were generated by aligning genomic DNA and mRNA sequences. The *S*_c_ haplotype and the associated FaSt MITE insertion within *SFB* were included to enable genotyping of *S*_c_-associated self-compatibility. Similarity searches, sequence editing, alignment, consensus construction and concatenation were performed with Geneious Prime 2024.0.3.

### Mapping to the synthetic *S*-locus reference and *S*-genotyping methodology

Individual shotgun sequencing reads from the 226 cultivated apricot accessions reported by Groppi et al^4^. were processed using a command-line *S*-locus genotyping workflow. Raw reads were first filtered against a *Prunus *S-locus database using the kmerRefFilter algorithm described by Genete et al^31^. To build the filtering database, sequences annotated in NCBI as *S-RNase* or *SFB*, including naming variants of these terms and belonging to *Prunus* species, were retrieved. These sequences were combined with the synthetic reference previously used for Japanese plum^36^ and the *P. armeniaca* synthetic reference generated in this study. Duplicate sequences were removed using SeqKit^59^. Filtered reads were trimmed with Trimmomatic^60^ and aligned to the synthetic S-locus reference using Bowtie2^61^ in end-to-end mode with the very-sensitive preset. Mapped reads were filtered with Samtools^62^ to retain only reads with mapping quality ≥ 20 and no more than two edit-distance differences relative to the reference sequence, as recorded by the NM tag (NM ≤ 2). Thus, reads with three or more differences were excluded. Bedtools^63^ was then used to calculate coverage statistics for each annotated reference gene.

Allele calls were based on Breadth of coverage, defined as the fraction of each reference gene covered by mapped reads. A minimum Breadth threshold of 0.50 was used for allele detection. This threshold was selected to distinguish true allele presence from background mapping and was supported by the distribution of Breadth values across all mappings, in which positive allele calls were separated from the large class of low-Breadth background signals (Supplementary Fig. S8). Because Breadth measures coverage continuity across the reference gene, localized read mapping to conserved or repetitive regions was insufficient to support genotype assignment.

For each accession, an R script was used to identify *S-RNase* and *SFB* genes with Breadth ≥ 0.50 and to summarize the corresponding *S*-genotype. Automated calls were complemented by visual inspection of BAM alignments in IGV^64^, particularly for ambiguous cases, borderline profiles, and the detection of self-compatibility associated with the *S*_c_ haplotype. For *S*_c_ genotyping, read continuity across the junction between the *SFB* coding region and the FaSt MITE insertion was inspected, because reads originating from dispersed genomic copies of the MITE can map to the insertion sequence without indicating the presence of the *S*_c_-associated *SFB* insertion.

To evaluate pipeline behavior when the synthetic reference was incomplete, selected *S*-locus genes were removed from the reference and accessions known to carry the removed alleles were remapped (Supplementary Fig. S9). The tested cases included removal of the *S*_13_/*SFB*_5_ haplotype, removal of the *S*_6_/*SFB*_6_ haplotype, and simultaneous removal of the *S*_4_/*SFB*_4_ and *S*_23_/*SFB*_23_ haplotypes. Breadth values were recalculated for the modified references and compared with mappings to the complete reference. This analysis was used to test whether absence of the correct reference allele resulted in false-positive reassignment to related alleles or in loss of a confident call. The R scripts used for automated allele calling, Breadth-threshold visualization, and reference-removal robustness analysis are available at: https://github.com/afifhedhly/apricot-s-locus-genotyping.

### PCR *S*-genotyping, cloning and Sanger sequencing

Total genomic DNA was extracted from 10 varieties (‘Henderson’, ‘Sunglo’, ‘Bulida’, ‘Canino’, ‘Fantasme’, ‘Flopria’, ‘Précoce de Tyrinthe’, ‘Currot’, ‘Murciana’, and ‘Lady Cot’), using the DNeasy Plant Mini Kit (Qiagen, Hilden, Germany), according to the manufacturer’s instructions. Different PCR reactions were performed to amplify either *SFB* or *S-RNase* alleles. Cultivars ‘Henderson’, ‘Sunglo’, and ‘Bulida’ were used to sequence the *S*_3_-RNase and *SFB*_5_ alleles for inclusion in the synthetic *S*-locus reference and to validate the NGS-based genotyping results for their remaining alleles. ‘Bulida’ and the remaining cultivars were used for *SFB* genotyping by PCR and gel electrophoresis and to clone and sequence a subset of PCR fragments. Different primer pair combinations commonly used in *Prunus* species or allele- and insertion-specific primer pairs were used for PCR and Sanger sequencing analyses (Fig. 1, Supplementary Figs. S5-S7, and Supplementary Table S4). PCR amplifications were performed using standard methods, and thermal cycling conditions were based on published studies^30,39,41^. Cloning was performed using the CloneJET PCR Cloning Kit with DH10B Competent Cells (Thermo Scientific, St Leon-Rot, Germany) according to the manufacturer’s instructions. Sequence analysis and similarity searches against the NCBI database were performed with Geneious Prime.

## Supplementary Information

Supplementary Information 1.

Supplementary Information 2.

## Data Availability

All datasets generated in the present study are provided in the Supplementary Materials of this article. No additional raw sequencing data were generated. Apricot reference genomes were downloaded from NCBI Datasets and the Genome Database for Rosaceae (GDR). The 226 whole-genome sequencing datasets are part of BioProject PRJEB42181 and were obtained from the ENA repository. All relevant information, including accession numbers for reference genomes and whole-genome sequencing datasets, is provided in the Methods section and Supplementary Materials. The R scripts used for automated allele calling, Breadth-threshold visualization, and reference-removal robustness analysis are available at https://github.com/afifhedhly/apricot-s-locus-genotyping.
