# Supplementary material for: A next-generation sequencing approach for high-resolution S-locus genotyping in apricot
Source: Sci Rep. 2026 Aug 26;16:26813. doi: 10.1038/s41598-026-59797-w (PMC13519023; doi:10.1038/s41598-026-59797-w)
Supplement: Supplementary file 1 [file 41598_2026_59797_MOESM2_ESM.pdf]

A next-generation sequencing approach for high-resolution S-locus genotyping in apricot.

Jorge Lora(0000-0001-9713-0431), Andrea Torres(0009-0004-2707-2730), José I. Hormaza(0000-0001-5449-7444), Javier Rodrigo(0000-0002-8321-1764) and Afif Hedhly(0000-0002-4761-3731)

Supplementary File 2: Fig. S1-S9

- Fig. S1: Maximum-likelihood phylogenetic tree of representative S-RNase allele groups reconstructed from the homologous C-terminal coding region used in Fig. 2A
- Fig. S2: Maximum-likelihood phylogenetic tree of representative SFB allele groups reconstructed from near-complete coding sequences in Fig. 2C
- Fig. S3: Additional maximum-likelihood phylogenetic analyses of S-RNase and SFB sequences using alternative alignments.
- Fig. S4: Structure of the synthetic S-locus reference sequence.
- Fig. S5: PCR genotyping of S_c-associated self-compatibility.
- Fig. S6: PCR fragment sequencing for S_c-associated self-compatibility.
- Fig. S7: Additional *SFB* and *S-RNases* sequenced in this study.
- Fig. S8: Distribution of Breadth values across all allele mappings.
- Fig. S9: Reference-removal robustness analysis.

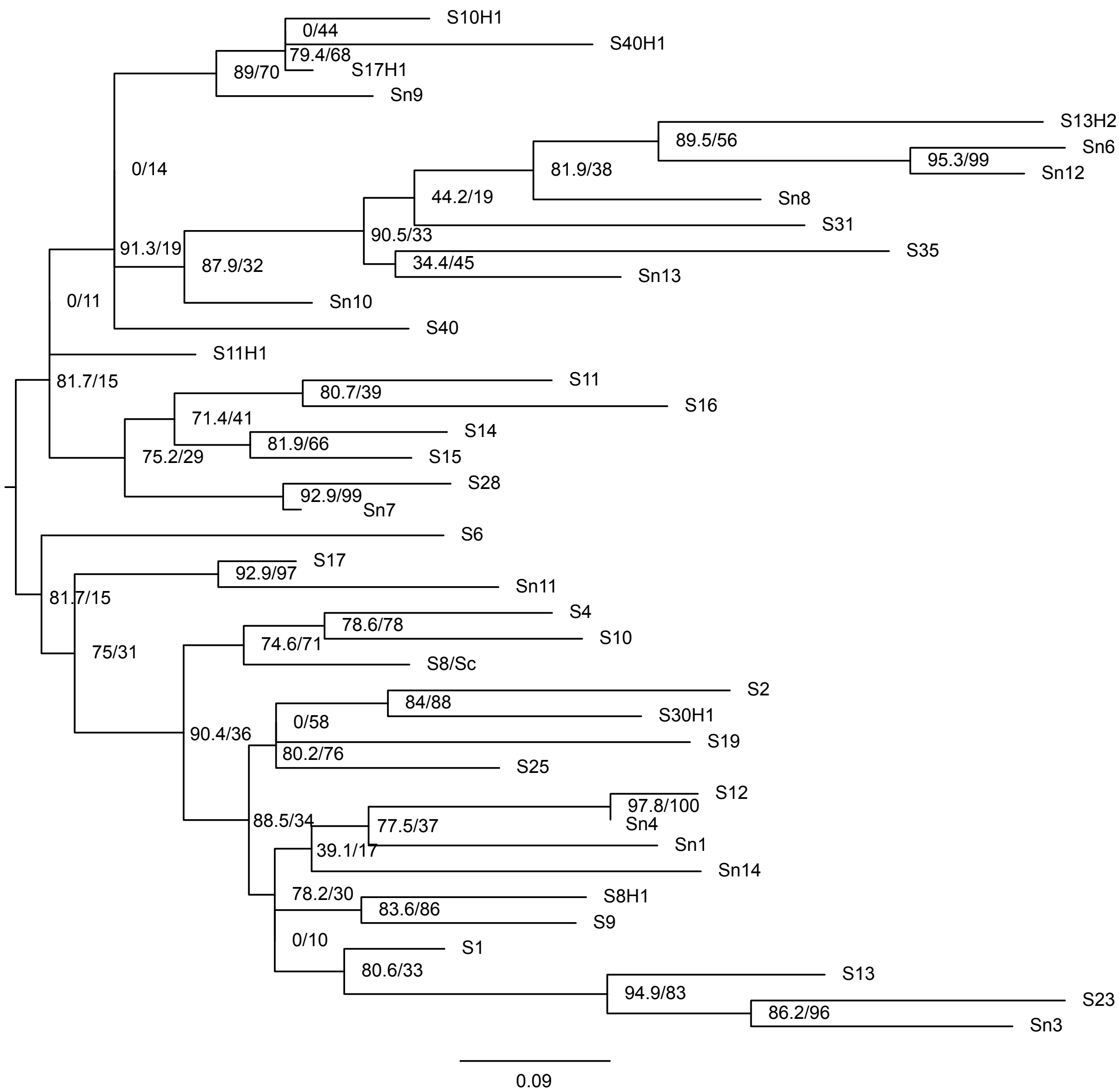

***Fig. S1** Maximum-likelihood phylogenetic tree of representative S-RNase allele groups reconstructed from the homologous C-terminal coding region used in Fig. 2A*. One representative sequence per allele group was included to summarize divergence among S-RNase allele groups and avoid overrepresentation of highly similar records. Branch values indicate SH-aLRT support (%) / ultrafast bootstrap support (%). The tree was midpoint-rooted for visualization.

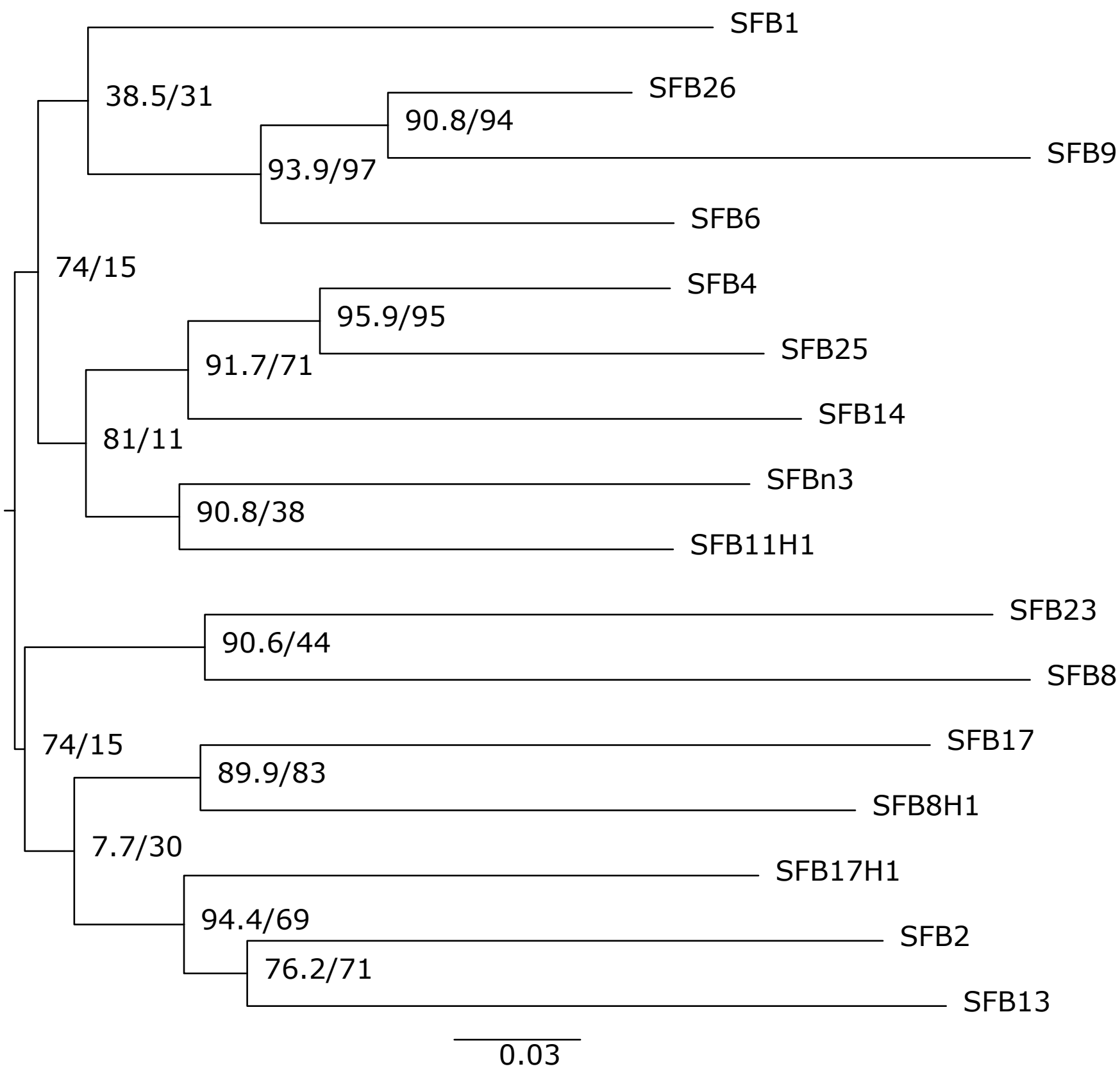

Fig. S2. *Maximum-likelihood phylogenetic tree of representative SFB allele groups reconstructed from near-complete coding sequences in Fig 2C.* One representative sequence per allele group was included to summarize divergence among SFB allele groups and avoid overrepresentation of highly similar records. The alignment includes the C-terminal variable and hypervariable regions where available, corresponding to the SFB tree summarized in Fig. 2C. Branch values indicate SH-aLRT support (%) / ultrafast bootstrap support (%). The tree was midpoint-rooted for visualization.

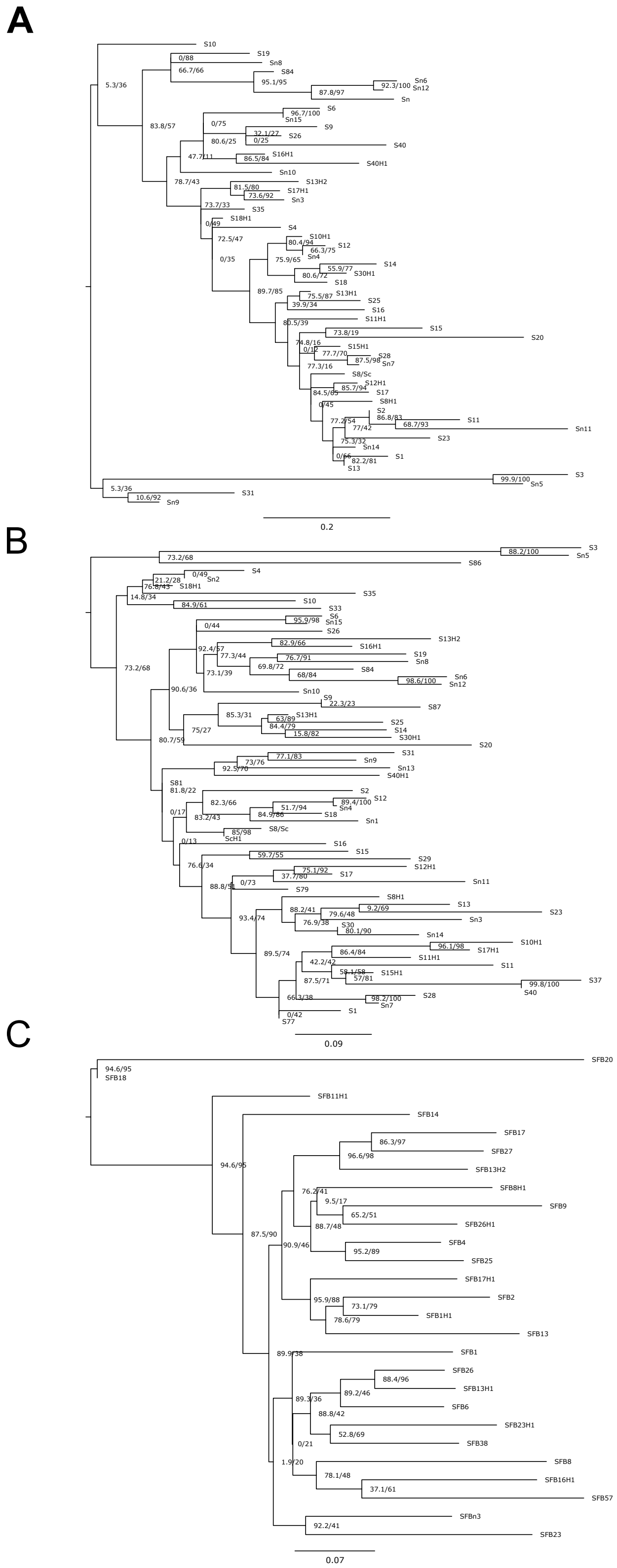

***Fig. S3** Additional maximum-likelihood phylogenetic analyses of S-RNase and SFB sequences using alternative alignments.* (A) S-RNase tree reconstructed from a short homologous region shared among available sequences. (B) S-RNase tree reconstructed from the broader amino-acid alignment including additional available sequences. (C) SFB tree reconstructed from the broader amino-acid alignment including shorter available SFB sequences. These analyses were used to evaluate the consistency of sequence relationships across alignments differing in length, sequence coverage and completeness. Branch values indicate SH-aLRT support (%) / ultrafast bootstrap support (%). Trees were midpoint-rooted for visualization. Because these analyses include partial and unevenly overlapping sequences, they are used as complementary summaries of sequence divergence rather than as fully resolved genealogies of S-locus alleles.

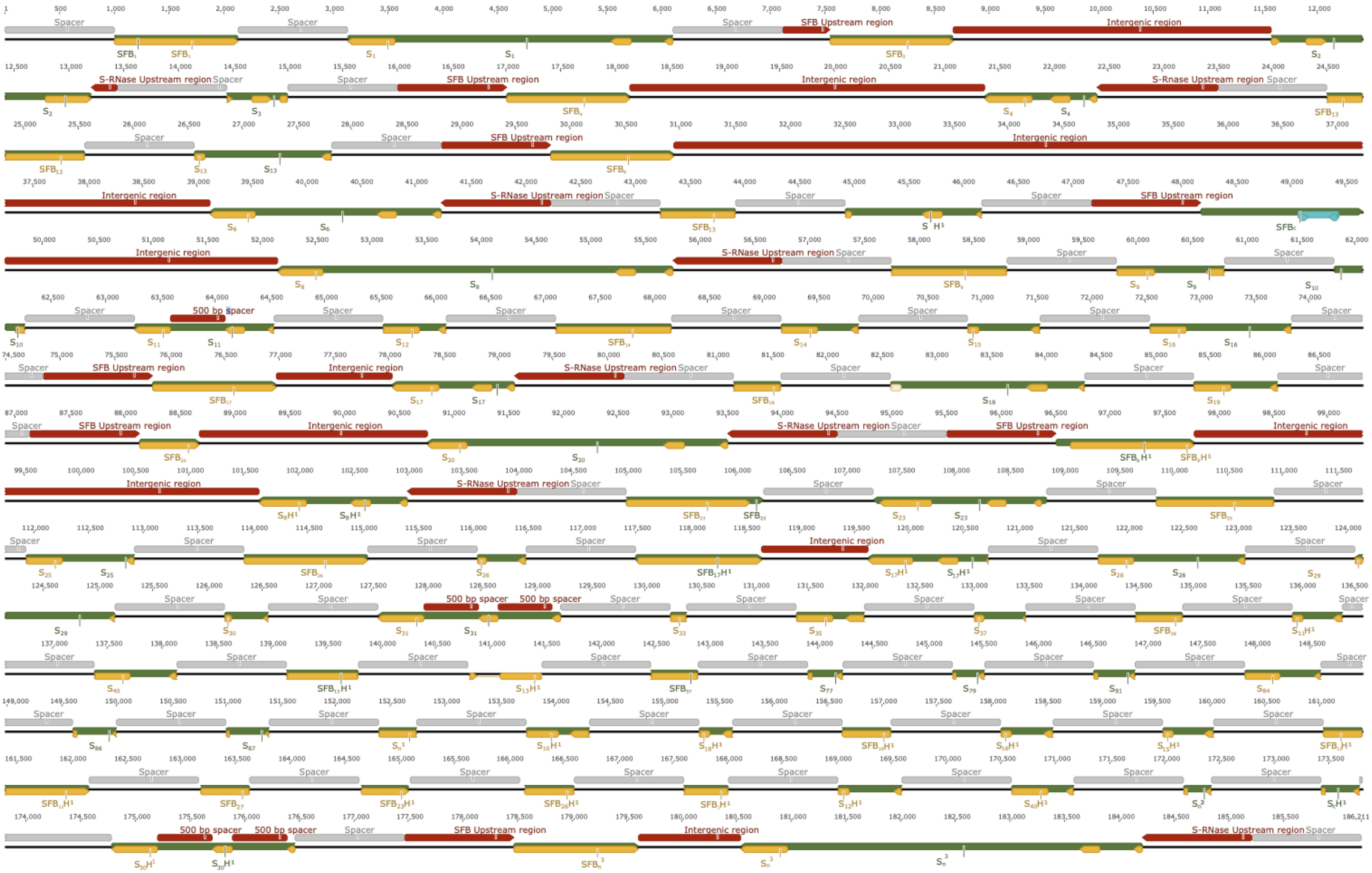

Fig. S4 *Structure of the synthetic S-locus reference sequence*. The synthetic reference was assembled by concatenating curated S-locus sequences, including complete S-haplotypes, individual S-RNase or SFB sequences, and orphan S-locus elements for which only one haplotype component was available. Grey boxes indicate spacer regions, yellow boxes indicate coding regions, green boxes indicate genes, red boxes indicate upstream, downstream and intergenic regions, and cyan indicates the FaSt MITE insertion associated with the Sc haplotype. The shape of each box indicates the orientation of the sequence element, and box length is proportional to sequence length. Accession information for the sequences included in the synthetic reference is provided in Table 1 and Supplementary Table S1.

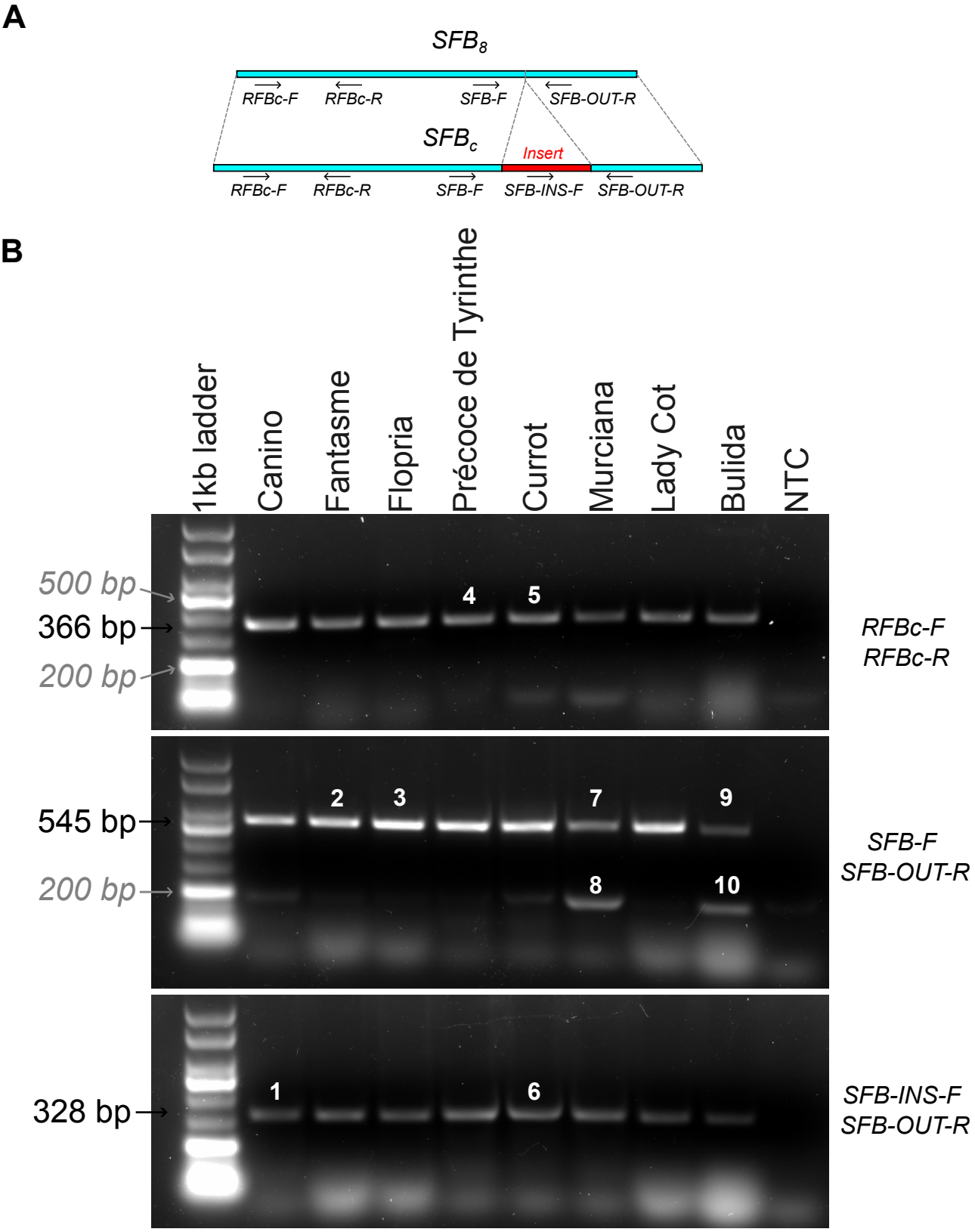

Fig. S5 PCR genotyping of S_c-associated self-compatibility. **A**) Schematic representation of the wild-type SFB8 sequence and the Sc-associated mutant SFB allele used for primer design; the FaSt insertion is highlighted in red. **B**) Eight varieties genotyped as carrying the Sc-associated SFB allele with our genotyping procedure were validated by PCR analysis. Three primer combinations were used: RFBc-F/RFBc-R, an SFB8-specific primer pair amplifying an expected fragment of approximately 366 bp outside the insertion site; SFB-F/SFB-OUT-R, which amplifies different fragments depending on whether the insertion is present or absent, approximately 545 bp and 200 bp, respectively; and SFB-INS-F/SFB-OUT-R, which produces a fragment only in the presence of the insertion, approximately 328 bp. The numbered PCR fragments were cloned and sequenced (see Fig. S6). NTC: non-template control.

Fig. S6. PCR fragment sequencing for Sc-associated self-compatibility. Sequences of PCR fragments amplified with the three primer combinations shown in Fig. S5. Sequence numbers correspond to the PCR fragment numbers indicated in Fig. S5. The transposon insertion sequence is highlighted in yellow.

>1_SFBc Canino_SFB-INS-F/SFB-OUT-R
ATATGGTAGTTTTGTAATTTCTAAGAAGTTAAAGCCTTTTTGTTATATTGTAAATGGGTTTTGGGTGTGTT
TATAAAGTTCATTTCATATAGGGTATTTTTATAATTTGGGTCTTCATATTGGGTATATTAGTAAATCTCCCTT
TTATTTATCCTCCGGATTATTATTATTGTACAACAGGGATTAGTATGGATAACAAAATCTTAATGCTAAGA
GTAGATGACATTAGAGGCATAAGAAATCTGGATTTATGTGATTACGAATCCAACCAAGTTCTTGAAACA
GGAATT

>1_SFBc Canino_SFB-INS-F/SFB-OUT-R_deduce_amino_acids_downstream_insert
IYPPDYYYCTTGISMDNKILMLRVDDIRGIRNLDLCDYESNQVLETGI

>2_SFBc Fantasme_SFB-F/SFB-OUT-R
GAGCCTTTTATTTAGGAGATATTTAGTGATATACCCATTCTTAACACTAATATTATAGATAAACCCTCCATC
CATTGAATTTCTATAAACAAACCCAAAAAAAACCCAAAAAATGACAACTGGCCTTATTGAATTTAATATT
GATTATTAAATTACTTTAATGCCCTATTGAGTGTTTTGGGTATTTTTATGAGATTTTGGGGTTGGGCTTGT
TTTAAGAAATTTATGGTAGTTTTGTAATTTCTAAGAAGTTAAAGCCTTTTTGTTATATTGTAAATGGGTTT
TGGGTGTGTTTATAAAGTTCATTTCATATAGGGTATTTTTATAATTTGGGTCTTCATATTGGGTATATTAGT
AAATCTCCCTTTTATTTATCCTCCGGATTATTATTATTGTACAACAGGGATTAGTATGGATAACAAAATCTT
AATGCTAAGAGTAGATGACATTAGAGGCATAAGAAATCTGGATTTATGTGATTACGAATCCAACCAAGT
TCTTGAAACAGGAATT

>2_SFBc Fantasme_SFB-F/SFB-OUT-R_deduce_amino_acids_upstream_downstream_insert
SLLIYPPDYYYCTTGISMDNKILMLRVDDIRGIRNLDLCDYESNQVLETGI

>3_SFBc Flopria_SFB-F/SFB-OUT-R
CAAATGAGTCTTTTAATTTAGAGATATTTAGTGATATACCCATTCTTAACACTAATATTATAGATAAACCCT
CCATCATTGAATTTCTATAAACAAACCCAAAAAAAACCCAAAAAATGACAACTGGCCTTATTGAATTTAA
TATTGATTATTAAATTACTTTAATGCCCTATTGAGTGTTTTGGGTATTTTTATGAGATTTTGGGGTTGGGC
TTGTTTTAAGAAATTTATGGTAGTTTTGTAATTTCTAAGAAGTTAAAGCCTTTTTGTTATATTGTAAATGG
GTTTTGGGTGTGTTTATAAAGTTCATTTCATATAGGGTATTTTTATAATTTGGGTCTTCATATTGGGTATAT
TAGTAAATCTCCCTTTTATTTATCCTCCGGATTATTATTATTGTACAACAGGGATTAGTATGGATAACAAAA
TCTTAATGCTAAGAGTAGATGACATTAGAGGCATAAGAAATCTGGATTTATGTGATTACGAATCCAACCA
AGTTCTTGAACCAAGGAAT

>3_SFBc_ Flopria_SFB-F/SFB-OUT-R_deduce_amino_acids_upstream_downstream_insert
QMSLLIYPPDYYYCTTGISMDNKILMLRVDDIRGIRNLDLCDYESNQVLEPRN

>4_SFB8 Precoce_de_Tyrinthe_RFBc-F/RFBc-FR
GGTTCAAGCAATGGTTTAGTTTGCATTTCGGATGAGATCCTGAATTTCGATAGTCCTATACACATATGGA
ACCCATCGGTTAGGAAATTTAGGGCCCTTCCAATGAGCACCAACATTAACATTAAATTTAGCTGTGTTG
CTCTCCAGTTCGGGTTCCACCCTGGGGTTAATGACTACAAGGCTGTAAGGATGATGCGTACCAATAAAA
GTGCCTTGGCGGTTGAGGTTTATAGTCTCAAAAGAGACTCTTGGAAGATGATTGAAGCAATTCCTCCT
TGGTTAAAATGCACTTTGGAACATCATAGGGGTA

>4_SFB8 Precoce_de_Tyrinthe_RFBc-F/RFBc-FR_deduced_amino_acids
GSSNGLVCISDEILNFDSPIHIWNPSVRKFRALPMSTNINIKFSCVALQFGFHPGVNDYKAVRMMRTNKS
ALAVEVYSLKRDSWKMIEAIPPWLKCTLEHHRG

>5_SFBc Currot_SFB-INS-F/SFB-OUT-R
TTATGGTAGTTTTGTAATTTCTAAGAAGTTAAAGCCTTTTTGTTATATTGTAAATGGGTTTTGGGTGTGTT
TATAAAGTTCATTTCATATAGGGTATTTTTATAATTTGGGTCTTCATATTGGGTATATTAGTAAATCTCCCTT
TTATTTATCCTCCGGATTATTATTATTGTACAACAGGGATTAGTATGGATAACAAAATCTTAATGCTAAGA
GTAGATGACATTAGAGGCATAAGAAATCTGGATTTATGTGATTACGAATCCAACCAAGTTCTTGAAACA
GGAATT

>5_SFBc_ Currot_SFB-INS-F/SFB-OUT-R_deduce_amino_acids_downstream_insert
IYPPDYYYCTTGISMDNKILMLRVDDIRGIRNLDLCDYESNQVLETGI

>6_SFB8 Currot_RFBc-F/RFBc-R
GGTTCAAGCAATGGTTTAGTTTGCATTTCGGATGAGATCCTGAATTTCGATAGTCCTATACACATATGGA
ACCCATCGGTTAGGAAATTTAGGGCCCTTCCAATGAGCACCAACATTAACATTAAATTTAGCTGTGTTG
CTCTCCAGTTCGGGTTCCACCCTGGGGTTAATGACTACAAGGCTGTAAGGATGATGCGTACCAATAAAA
GTGCCTTGGCGGTTGAGGTTTATAGTCTCAAAAGAGACTCTTGGAAGATGATTGAAGCAATTCCTCCT
TGGTTAAAATGCACTTTGGAACATCATAGGGGTA

>6_SFB8_ Currot_RFBc-F/RFBc-R_deduce_amino_acids_upstream_insert
GSSNGLVCISDEILNFDSPIHIWNPSVRKFRALPMSTNINIKFSCVALQFGFHPGVNDYKAVRMMRTNKS
ALAVEVYSLKRDSWKMIEAIPPWLKCTLEHHRG

>7_SFBc Murciana_SFB-F/SFB-OUT-R
CCAAATGAGTCTTTTATTTAGAGATATTTAGTGATATACCCATTCTTAACACTAATATTATAGATAAACCCT
CCATCATTGAATTTCTATAAACAAACCCAAAAAAAACCCAAAAAATGACAACTGGCCTTATTGAATTTAA
TATTGATTATTAAATTACTTTAATGCCCTATTGAGTGTTTTGGGTATTTTTATGAGATTTTGGGGTTGGGC
TTGTTTTAAGAAATTTATGGTAGTTTTGTAATTTCTAAGAAGTTAAAGCCTTTTTGTTATATTGTAAATGG
GTTTTGGGTGTGTTTATAAAGTTCATTTCATATAGGGTATTTTTATAATTTGGGTCTTCATATTGGGTATAT
TAGTAAATCTCCCTTTTATTTATCCTCCGGATTATTATTATTGTACAACAGGGATTAGTATGGATAACAAAA
TCTTAATGCTAAGAGTAGATGACATTAGAGGCATAAGAAATCTGGATTTATGTGATTACGAATCCAACCA
AGTTCTTGAA

>7_SFBc Murciana_SFB-F/SFB-OUT-R_deduce_amino_acids_upstream_downstream_insert
PNESFIYPPDYYYCTTGISMDNKILMLRVDDIRGIRNLDLCDYESNQVLE

>8_SFB6 Murciana_SFB-F/SFB-OUT-R
TGGGTTCTGCAAGAAAAACGGTGGAAACAATTGTGTCCTTTTATCCATCCTTTTGATTGTGATGGTACA
ATCGGGATTAGTATTGATAATGAACTCCTAATAGAAAGAAGAGATTTCGATAGGGGTGTAGCAG

>8_SFB6 Murciana_SFB-F/SFB-OUT-R_deduced_amino_acids
WVLQEKRWKQLCPFIHPFDCDGTIGISIDNELLIERRDFDRGVA

>9_SFBc Bulida_SFB-F/SFB-OUT-R

ATTTAGAGATATTTAGTGATATACCCATTCTTAACACTAATATTATAGATAAACCCTCCATCATTGAATTTCT
ATAAACAAACCCAAAAAAAACCCAAAAAATGACAACTGGCCTTATTGAATTTAATATTGATTATTAAATT
ACTTTAATGCCCTATTGAGTGTTTTGGGTATTTTTATGAGATTTTGGGGTTGGGCTTGTTTTAAGAAATT
TATGGTAGTTTTGTAATTTCTAAGAAGTTAAAGCCTTTTTGTTATATTGTAAATGGGTTTTGGGTGTGTTT
ATAAAGTTCATTTCATATAGGGTATTTTTATAATTTGGGTCTTCATATTGGGTATATTAGTAAATCTCCCTTT
TATTTATCCTCCGGATTATTATTATTGTACAACAGGGATTAGTATGGATAACAAAATCTTAATGCTAAGAG
TAGATGACATTAGAGGCATAAGAAATCTGGATTTATGTGATTACGAATCCAACCAAGTTCTTGAAACAG
GAATTA

>9_SFBc_ Bulida_SFB-F/SFB-OUT-R_deduce_amino_acids_downstream_insert
IYPPDYYYCTTGISMDNKILMLRVDDIRGIRNLDLCDYESNQVLETGI

>10_SFB5 Bulida_SFB-F/SFB-OUT-R
CTTTATCCTTCGGATTATTATTATCGTACAATCGGGATTAGCATAGATAGTAAAATCTTAATGCTAAGAGC
AGATCACATTAGGGGCATATTAGATATGCATTTGTGTGATTGCGAATCCAAGCAAGTTCTTGAAACAGG
AATTA

>10_SFB5 Bulida_SFB-F/SFB-OUT-R_deduced_amino_acids
YPSDYYYRTIGISIDSKILMLRADHIRGILDMHLCDCESKQVLETGI

Fig. S7. Additional SFB and S-RNase sequences obtained in this study. Nucleotide and deduced amino acid sequences of cloned PCR fragments amplified with commonly used primers in Prunus species. Primer pairs are specified in the sequence descriptions. The transposon insertion sequence associated with S_c is highlighted in yellow.

>12_S-RNase_S_8_first_intron_Murciana_SRcF/R
TGAGCACTAGTGGTGGGTTGCATTACAATCTTTTGCTCTTTATATCATATATGCATATAATTAGAATTACGA
AGGAGAAGTAGGCAGGAAATGTCATTAATTAGTACATAACTTTCTTTGGATGAGTTACTATTTGGGAAT
TATTTTTCTGCATGGTATCTTTCGATTACTCTGATAGTTGTTGAAATAAGTGCAGTATTCATCATTGGAAG
CTAAAATGGTGTTCTTCCTGCATAAAATCCATTAACCTTCTCACAATAATTTTCGCAGGATCTTATGTCTAT
TTTCAATTTGTGCAACAATGGC

>12_S-RNase_ S_8_first_intron_Murciana_SRcF/R_deduced_amino_acids
STRSYVYFQFVQQW

>13_S-RNase_S9 Henderson_PruC2/C4R
CTATGGCCAAGTAATTATTCAAACCCAAGGTTGCCCAGTAATTGCATTGGGTCGCAATTTAAGGGAATA
TTGGTATGTATTGGTTTTGTTTTTCACATGCTCTTTAGCTTTTAGTTTTTACAAAATTTGATTGTCATATGG
AGCTAATAAATTTTTTCACTAAACCTTGGCTTTTAGGTAAAAGTTTGATGTTGGTCCTTTGGTTCGACAC
ACTTGAAAATGTATCTAAGCACGAAATTGTATATTTATATTATCCATGTATATATTTATAATATTGGATTTGA
TGATCTAATTAAGGGACTTAAAATGTTGTACTTATGTATATATATTCAAAAATTGTGCTCACTGAAAGGAT
ATCAAGGTCAAAATTCAATTTAATACTCAGGCTTAATGAAAAAAAAATCTTATCTAAAAATGAAAATGTA
ACTATCCCTAAAGGTTGTACTTTTTTCTTTGGCCAAAAAATGAAAAAAGGCTTTAACTTTTTTTCTCAAA
CTATGTCTTCATTTTTTTTTCCTCAAATATTTGCTTGGATGTCTCAGTCCCCTCAATTGCGGTCCAAACTG
AAGACATCATGGCCGGACGTGGAAGGTGGCAATGATACAAAGTTTTGGGAAGGCGAATGGAACAAA
CACGGTACATGTTCCGAACATACACTTAACCAAATGCAGTACTTCGAGCGATCCTACGCAATGTGGATG
TCGTACAATATTACAGGGGTCCTTAAAAACGCTTCAATCGTACCA

>13_S-RNase_S_9_Henderson_PruC2/C4R_deduced_amino_acids
LWPSNYSNPRLPSNCIGSQFKGILVSPQLRSKLKTSWPDVEGGNDTKFWEGEWNKHGTCSEHTLNQMQ
YFERSYAMWMSYNITGVLKNASIVP

>14_S-RNase_S_3_Henderson_PruC2/C4R
GCTTTCCTTGTTCTTGCTTTGGTCTTGTTCTTGTGTTTCAGTATGAGCACTGGTGGGTTGCATTATAAATA
TTTGTGCTCGATGGTATATGTACTTTGTTATTTATTACATTACCATTGCGTTTCTACTTGTATTTTCACTCGG
ATCAAGTAACTATTTGTTTGGGAATTATTTTCTACATTGTTACTTTTGTGTTTCAGTAAGCTAAAATTATGT
TCTTTATGCAGGATCTTATCAATATTTTCAGTTTGTGCAACAATGGCCGCCAACCACCTGCGCAATTAGC
AAAAAACCTTGCTACCAAAACCCGCCCTCAATCTTCACCATCCATGGCCTATGGCCAAGTAATCATTCAA
AAAACGCATGGGTGGCTAATTGCAGTCCAACGCGATTTAACAATAGTTTGGTACGGACTGTTTTGTTTT
TCTAGTTAGTCTTTAGTTTTTGTATGTTCGTCACAATATATTTTATTGCTTGCATGTCTCAGTCTCCTAGAT
TGGAAACCAAACTGAAGATATCTTGGCCCAACGTGGAAAGTGGCAATTATACAGACTTTTGGGAA

>14_S-RNase_S_3_Henderson_PruC2/C4R_deduced_amino_acids
AFLVLALVLFLCFSMSTGSYQYFQFVQQWPPTTCAISKKPCYQNPPSIFTIHGLWPSNHSKNAWVANCSPT
RFNNSLSPRLETKLKISWPNVESGNYTDFWE

>15_S-RNase_S_3_Sunglo_PruC2/C4R

GCTTTCCTTGTTCTTGCTTTGGTCTTGTTCTTGTGTTTCAGTATGAGCACTGGTGGGTTGCATTATAAATA
TTTGTGCTCGATGGTATATGTACTTTGTTATTTATTACATTACCATTGCGTTTCTACTTGTATTTTCACTCGG
ATCAAGTAACTATTTGTTTGGGAATTATTTTCTACATTGTTACTTTTGTGTTTCAGTAAGCTAAAATTATGT
TCTTTATGCAGGATCTTATCAATATTTTCAGTTTGTGCAACAATGGCCGCCAACCACCTGCGCAATTAGC
AAAAAACCTTGCTACCAAAACCCGCCCTCAATCTTCACCATCCATGGCCTATGGCCAAGTAATCATTCAA
AAAACGCATGGGTGGCTAATTGCAGTCCAACGCGATTTAACAATAGTTTGGTACGGACTGTTTTGTTTT
TCTAGTTAGTCTTTAGTTTTTGTATGTTCGTCACAATATATTTTATTGCTTGCATGTCTCAGTCTCCTAGAT
TGGAAACCAAACTGAAGATATCTTGGCCCAACGTGGAAAGTGGCAATTATACAGACTTTTGGGAA

>15_S-RNase_S_3_Sunglo_PruC2/C4R_deduced_amino_acids
AFLVLALVLFLCFSMSTGSYQYFQFVQQWPPTTCAISKKPCYQNPPSIFTIHGLWPSNHSKNAWVANCSPT
RFNNSLSPRLETKLKISWPNVESGNYTDFWE

>16_S-RNase_S_2_first_intron_Sunglo_SRcF/PCER
CTCGCTTTCCTTGTTCTTGCTTTCTTCTTGTGTTTCATAATGAGCACTGGTGATGGTGGGTTGCATTACA
ATCTTTTGCTCTTTATATGCCATGTATGTGCATATAATTTGCATTGAATTTTTCTACTTCTATTTTATGTGTG
GATAACTATTGTATGTTTTCGATGATAGGGAGGACTTATATAGCGCACAACTTTCTTTACTCTGATAGTGT
TGCAATAAGTACAGTATTCATCATTGGAAACCTTATTGAAGATACCATTAACCTTTTATCACAATAATTTT
GGCAGGAACTTATGACTATTTCCAATTTGTGCAACAATGGCCACCGACCACCTGCGGAGTTCGCGGGA
AACCTTGCTCCAAACCCCGGCTATTACAAAATTTCACCATCCATGGCCTATGGCCAAGTAATTATTCAAA
CCCAACGATGCCTAGTAATTGCAATGGGTCGAAATTTGAGGCAAGGAAAGTGGTATGTATTGTTTTTAT
TTATTTATTTATTAATAAATGTATATATAGGTATACCTATGTATAGATCCCTTTATAATAATGTATGTATCATCT
AATTAAAGGCATATAAATTTTCCTGCAACGGAATGCTTGGTTAGCTCTTTATATAATTATCCCCTTCCAATA
AGATGGTGCCATCACCATTTCATACAAATTTTTCTACTTAAAAACCTCCTAGTTGTTGGATATGGGTTTAC
AAATGGCACCATCTTATTGGAAAGGAGATGATTATGTGGAGAGCTGACCTGGCATTCCCGTGCCCATG
AAAGTTTCTATCACTTTAGAGGAGAGAAAGTAGGTATGGCAATGGAACATCACTTAGGGTGTGTTCCG
GTTAGAGATATAACCGAACACCATGCTCGAAAATTTTCCGCAAACTCATCCGTCCTTATATTCGACTTCTA
GTTTTTATAACCCATTAGATCGAAGCCCTGCAACTCGTGGAGAAATTTGTCATCCCTAGGAAAAAGGGA
TAACCTATCATTTTGTACTTACGTAGACATTCAGTATTTAAAATATACTATTTATGAAAGGATCAAAATCAA
AAGTCAATTTAATACTCAGGTTCA

>16_S-RNase_S_2_first_intron_Sunglo_SRcF/PCER_deduced_amino_acids
LAFLVLAFFLCFIMSTGDGTYDYFQFVQQWPPTTCGVRGKPCSKPRLLQNFTIHGLWPSNYSNPTMPSNC
NGSKFEARKV

>17_S-RNase_S_2_second_intron_Sunglo_Pru C2/PCER
CTATGGCCAAGTAATTATTCAAACCCAACGATGCCTAGTAATTGCAATGGGTCGAAATTTGAGGCAAGG
AAAGTGGTATGTATTGTTTTTATTTGTTTATTTATTAATAAATGTATATATAGGTATACCTATGTATAGATCCC
TTTATAATAATGTATGTATCATCTAATTAAAGGCATATAAATTTTCCTGCAACGGAATGCTTGGTTAGCTCT
TTATATAATTATCCCCTTCCAATAAGATGGTGCCATCACCATTTCATACAAATTTTTCTACTTAAAAACCTC
CTAGTTGTTGGATATGGGTTTACAAATGGCACCATCTTATTGGAAAGGAGATGATTATGTGGAGAGCAG
ACCTGGCATTCCCGTGCCCATGAAAGTTTCTATCACTTTAGAGGAGAGAAAGTAGGTATGGCAATGGA
ACATCACTTAGGGTGTGTTCCGGTTAGAGATATAACCGAACACCATGCTCGAAAATTTTCCGCAAACTC
ATCCGTCCTTATATTCGACTTCTAGTTTTTATAACCCATTAGATCGAAGCCCTGCAACTCGTGGAGAAATT
TGTCATCCCTAGGAAAAAGGGATAACCTATCATTTTGTACTTACGTAGACATTCAGTATTTAAAATATACT
ATTTATGAAAGGATCAAAATCAAAAGTCAATTTAATACTCAGGTTCAATGGGAAAAAGAGATCCAAGA
ATGAAAGTCTAATCATTCCTTTAGCTTTACTCTTTTTTATTTAGGAATTTCTGAATAAATATTGCTTGTATG

ACTCAGTACCCTCAATTGCGATCCGATCTGAAGATATCTTGGCCGGACGTGGAAAGTGGCAATGACAC
AAAATTTTGGGAAGGCGAATGGAACAAACA

>17_S-RNase_S_2_second_intron _Sunglo_Pru C2/PCER_deduced_amino_acids
WPSNYSNPTMPSNCNGSKFEARKVYPQLRSDLKISWPDVESGNDTKFWEGEWNK

>18_SFB_c Bulida_PsSFBF/R
GAAATCTTGATCGACGTCCTAGCAAGACTACCTGCAAAATCCCTTATTCGCTTTCTGAGTACATGCAAGT
CGTGGAGTGATTTGATTGGAAGCTCAATTTTTGTTAGCACACACCTTTGTAGGAATGTGACAAAACATA
CCCATGTTTATCTACTTTGCCTCCACCACCCAAATTTTGAGCGTTTGGTCGACCCTAATGACCCATATCTT
AAAAAGGAATTTCAATGGTCTCTTTTTCCCAAAGAAACATTTGAGGAGTGCTACAAACTAAGCCATCCC
TTAGGGATGACAGAACATTATGGGATATATGGTTCAAGCAATGGTTTAGTTTGCATTTCGGATGAGATC
CTGAATTTCGATAGTCCTATACACATATGGAACCCATCGGTTAGGAAATTTAGGGCCCTTCCAATGAGCA
CCAACATTAACATTAAATTTAGCTGTGTTGCTCTCCAGTTCGGGTTCCACCCTGGGGTTAATGACTACAA
GGCTGTAAGGATGATGCGTACCAATAAAAGTGCCTTGGCGGTTGAGGTTTATAGTCTCAAAAGAGACT
CTTGGAAGATGATTGAAGCAATTCCTCCTTGGTTAAAATGCACTTTGGAACATCATAGGGGTACGTTTT
TCAGTGGAGTAGCATACCACATCATTCAGAAAGGTCCTATGCTCAGCATTATGTCATTCGATTCAGGCAG
TGAAAAATTCGAAGAAATCATAGCACCAGATGCCATTTGCAGTTTATGGGGGTTATATATTGACGTTTAC
AAGGAACAAATTTGCTTGCTTTTTATATGTTATGGGTGTGAGGAGGAGGGCATGGAAAAAGCTGACTT
ATGGGTTCTGCAAGAAAAACGGTGGAAACAATTGAGTCCTTTTATTTAGAGATATTTAGTGATATACCC
ATTCTTAACACTAATATTATAGATAAACCCTCCATCATTGAATTTCTATAAACAAACCCAAAAAAAACCCA
AAAAATGACAACTGGCCTTATTGAATTTAATATTGATTATTAAATTACTTTAATGCCCTATTGAGTGTTTT
GGGTATTTTTATGAGATTTTGGGGTTGGGCTTGTTTTAAGAAATTTATGGAAGTTTTGTAATTTCTAAGA
AGTTAAAGCCTTTTTGTTAAATGGAAATGGGTTTTGGGGGTGTTATAAAGCTCATTTCATATAGGGGAT
TTTTATAATTGGGGGCTTCAATTGGGGAAATAAAAAAATCCCCCTTTTATTAACCCCCGAATAATATTT

>11_SFB_c Bulida_PsSFBF/R_deduced_amino_acids (up_to_the_insert)
EILIDVLARLPAKSLIRFLSTCKSWSDLIGSSIFVSTHLCRNVTKHTHVYLLCLHHPNFERLVDPNDPYLKKEF
QWSLFPKETFEECYKLSHPLGMTEHYGIYGSSNGLVCISDEILNFDSPIHIWNPSVRKFRALPMSTNINIKFS
CVALQFGFHPGVNDYKAVRMMRTNKSALAVEVYSLKRDSWKMIEAIPPWLKCTLEHHRGTFFSGVAYHII
QKGPMLSIMSFDSGSEKFEEIIAPDAICSLWGLYIDVYKEQICLLFICYGCEEEGMEKADLWVLQEKRWKQL
SPFINPRIIF

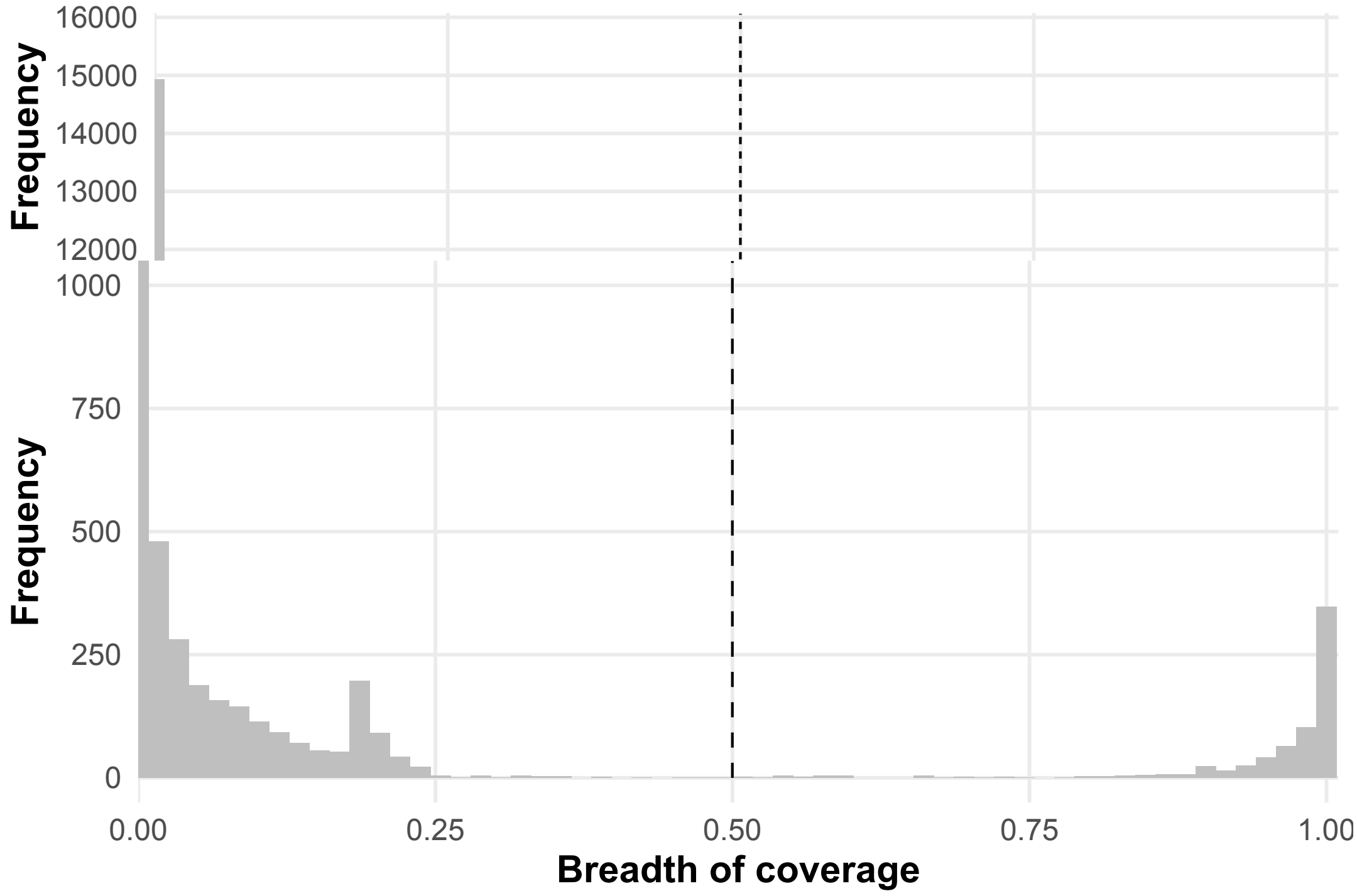

Fig. S8 *Distribution of Breadth values across all allele mappings.* Breadth was calculated as the fraction of each reference gene covered by mapped reads. Most mappings showed very low Breadth values, corresponding to background or non-specific alignments, whereas positive allele calls clustered at high Breadth values, frequently near complete coverage. The dashed vertical line indicates the 0.50 Breadth threshold used for allele calling. The y-axis is manually split to visualize both the large number of near-zero Breadth values and the lower-frequency high-Breadth values.

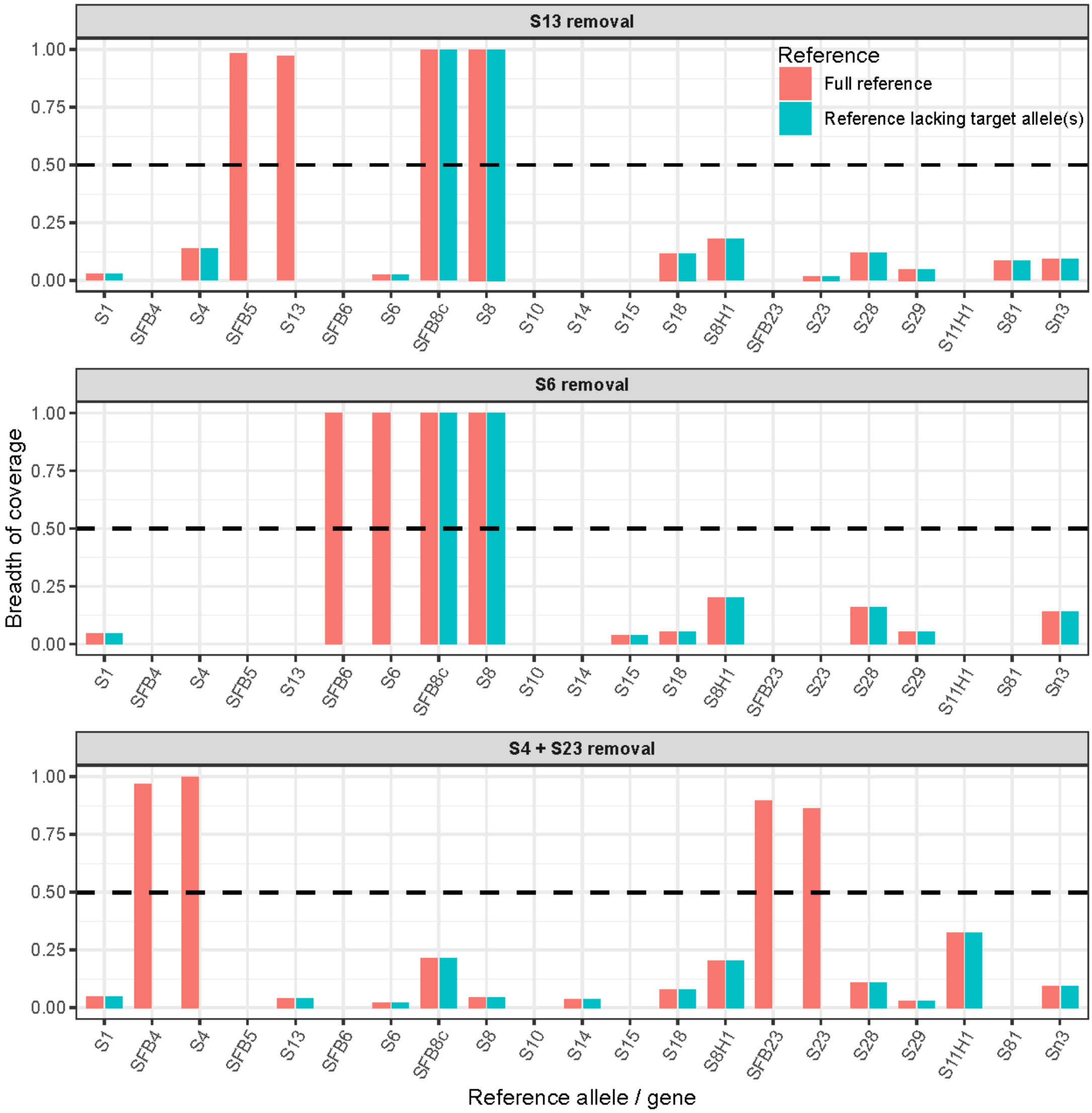

Fig. S9 Reference-removal robustness analysis.
Breadth of coverage values are shown for three representative accessions mapped against the complete synthetic reference sequence and against modified references lacking selected target allele(s). Dashed horizontal lines indicate the 0.50 Breadth threshold used for confident allele calling. Removal of the S13/SFB5 haplotype, the S6/SFB6 haplotype, or the combined S4/SFB4 and S23/SFB23 haplotypes eliminated the corresponding high-Breadth signals. Retained true alleles remained confidently detected, whereas residual signals at other alleles remained below the calling threshold. These results indicate that when the correct allele is absent from the reference, the pipeline does not generate a false-positive replacement call but instead produces a pattern consistent with an unrepresented allele.
